# Optimization, gene cloning, expression, and molecular docking insights for enhanced cellulase enzyme production by *Bacillus amyloliquefaciens* strain elh1

**DOI:** 10.1186/s12934-024-02454-6

**Published:** 2024-07-02

**Authors:** Elham F. El-Khamisi, Effat A. M. Soliman, Ghada M. El-Sayed, Shaimaa A. Nour, Mohamed O. Abdel-Monem, Mervat G. Hassan

**Affiliations:** 1 Microbial Genetics Department, Biotechnology Research Institute, National Research Centre, 33 El-Bohouth St., (Former El-Tahrir St.) Dokki, P.O. 12622, Giza, Egypt; 2 Chemistry of Natural and Microbial Products Department, Pharmaceutical and Drug Industries Research Institute, National Research Centre, 33 El-Bohouth St., Dokki, P.O. 12622, Giza, Egypt; 3 Botany and Microbiology Department, Faculty of Science, Benha University, Benha, 13511 Egypt

**Keywords:** Cellulase, *Bacillus amyloliquefaciens*, Optimization, Gene cloning and expression, Molecular docking

## Abstract

**Background:**

In this study, we isolated a cellulase-producing bacterium, *Bacillus amyloliquefaciens* strain elh, from rice peel. We employed two optimization methods to enhance the yield of cellulase. Firstly, we utilized a one-variable-at-a-time (OVAT) approach to evaluate the impact of individual physical and chemical parameters. Subsequently, we employed response surface methodology (RSM) to investigate the interactions among these factors. We heterologously expressed the cellulase encoding gene using a cloning vectorin *E. coli* DH5α. Moreover, we conducted in silico molecular docking analysis to analyze the interaction between cellulase and carboxymethyl cellulose as a substrate.

**Results:**

The bacterial isolate eh1 exhibited an initial cellulase activity of 0.141 ± 0.077 U/ml when cultured in a specific medium, namely Basic Liquid Media (BLM), with rice peel as a substrate. This strain was identified as *Bacillus amyloliquefaciens* strain elh1 through 16S rRNA sequencing, assigned the accession number OR920278 in GenBank. The optimal incubation time was found to be 72 h of fermentation. Urea was identified as the most suitable nitrogen source, and dextrose as the optimal sugar, resulting in a production increase to 5.04 ± 0.120 U/ml. The peak activity of cellulase reached 14.04 ± 0.42 U/ml utilizing statistical optimization using Response Surface Methodology (RSM). This process comprised an initial screening utilizing the Plackett–Burman design and further refinement employing the BOX -Behnken Design. The gene responsible for cellulase production, egl, was effectively cloned and expressed in *E. coli* DH5α. The transformed cells exhibited a cellulase activity of 22.3 ± 0.24 U/ml. The egl gene sequence was deposited in GenBank with the accession number PP194445. In silico molecular docking revealed that the two hydroxyl groups of carboxymethyl cellulose bind to the residues of Glu169 inside the binding pocket of the CMCase. This interaction forms two hydrogen bonds, with an affinity score of −5.71.

**Conclusions:**

Optimization of cultural conditions significantly enhances the yield of cellulase enzyme when compared to unoptimized culturing conditions. Additionally, heterologous expression of egl gene showed that the recombinant form of the cellulase is active and that a valid expression system can contribute to a better yield of the enzyme.

## Background

Agricultural and industrial wastes are major contributors to environmental pollution. However, if we transform them into valuable resources, we can reduce their negative impacts. Common examples of these wastes in many countries, such as leaves, straws, cereals, and corncobs, are often used as animal feed [1]. Unfortunately, a significant amount of these materials is left on farmlands to decompose naturally with the help of microorganisms like bacteria and fungi, which produce cellulase enzymes responsible for the biodegradation of cellulose, the major constituent of agricultural waste [2]. Both cultivated and uncultivated bacteria can yield cellulases. Microbial cellulases are obtained from cultivated microorganisms that can be isolated in a laboratory. Numerous microorganisms produce cellulases, including bacteria, fungi, and actinomycetes. Conversely, the cultivation-independent approach, which relies on uncultured microorganisms, faces limitations due to the inability to cultivate the majority of microorganisms, especially those inhabiting soil environments, in laboratory settings [3]. Bacterial cellulases have gained more attention than their fungal counterparts for several reasons. Bacterial cellulases are preferred due to their lower production costs compared to fungal cellulases [4, 5]. Moreover, bacteria have faster growth rates than fungi, enabling them to reach high cell densities more quickly. This, in turn, facilitates efficient enzyme production. Additionally, certain bacterial cellulases are expressed in multiple complexes, which enhance overall performance through synergistic effects [6]. Most researchers are currently focusing on scaling up the production of a vital enzyme for the industry by using diverse bacterial strains. However, there is significant variability among these enzymes in terms of molecular weight, stability, amino acid composition, classification into protein families and domains, and secondary and tertiary structures [6, 7]. To address this complexity, bioinformatics—an interdisciplinary field—is employed to analyze the structure and function of proteins using various computational tools and databases. The information obtained from these tools and databases can help select the most efficient bacterial strains for industrial enzyme production. Additionally, this information can be used to guide the development of new microbial strains with enhanced enzyme production capabilities through the application of recombinant DNA technology [8, 9]. A breakthrough in cellulase research occurred in 1982 when the first cellulase gene was successfully cloned [10]. Since then, numerous cellulase genes have been engineered for expression in different host organisms, including *Escherichia coli*, *Saccharomyces cerevisiae*, and *Pichia pastoris* [11–13]. Each organism exhibited varying levels of enzymatic activity. For example, the Cellobiohydrolase I (CBHI) gene, obtained from *Trichoderma koningii*, was integrated into the pGAPZ A plasmid and introduced into *Pichia pastoris*, which exhibited significant activity, reaching 0.1276 U/ml in the supernatant [14]. In another example, the gene encoding cellobiohydrolase was isolated from the *Clostridium clariflavum* and was adequately heterologous expressed in *Escherichia coli* BL21 (DE3). Through careful optimization of parameters such as induction time, pH, isopropyl β-d-1-thiogalactopyranoside (IPTG) concentration, and temperature, the highest enzyme activity achieved was 2.78 U /ml [15].

The core components of cellulase, which include endoglucanases (EC 3.2.1.4), exoglucanases (EC 3.2.1.74), and β-glucosidase (EC 3.2.1.21), play vital roles in fully breaking down cellulose [15, 16]. Cellulase is the second most important enzyme after amylase and is crucial for environmentally friendly and economically viable biofuel development [17]. Since the 1960s, cellulase has been increasingly used in various industries such as food, paper, pulp, textiles, and pharmaceuticals [18]. Specifically, cellulase (E.C. 3.2.1.4) is essential for breaking down lignocellulosic materials into simpler sugars, demonstrating its versatility in fields such as alternative energy, textiles, detergents, livestock feed, pharmaceuticals, food production, nutrition, and agriculture [19]. While the conventional chemical breakdown of cellulose is simple, the enzymatic process is distinctive for its lack of pollution, cost-effectiveness, and economic viability. Given these advantages, industries are increasingly looking for cost-effective cellulase with versatile applications, leading to the exploration of microorganisms capable of efficiently producing cellulase [20]. This pursuit opens up new opportunities for the discovery of economically viable cellulase-producing microorganisms. Numerous factors can be adjusted to enhance enzyme productivity and yield, including media components such as carbon, nitrogen, mineral sources, additives, and inducers, as well as physical parameters like pH, aeration, and temperature. These factors play a crucial role in determining the cost of enzyme production, which is often considered a primary challenge in biotechnological processes [21]. Researchers typically use a one-variable-at-a-time (OVAT) approach to screen physical and chemical parameters. The aim is to identify optimal conditions and understand the specific impact of each variable. However, this method is time-consuming and may overlook interactions between variables, leading to suboptimal results [21, 22].

To address these challenges, investigational methods based on factorial designs coupled with statistics have been developed and utilized to achieve faster and more reliable outcomes. Among these methods, the two-level factorial model is particularly advantageous as it facilitates the analysis of interactions between factors. The Plackett–Burman design (PBD) is one factorial-based statistical technique used to assess the significance of critical variables. Additionally, response surface methodology (RSM) is employed to study the interactions among independent process variables [23].

The computational methods rely on diverse structural and physicochemical properties of protein sequences to extensively analyze and characterize, providing a thorough understanding of the connections between function, structure, and interactions with substrates. To tackle the challenge posed by this complexity, the most reliable and precise approach is considered to be template-based modeling. By using computational techniques, numerous models have been developed and evaluated, specifically revealing the intricate protein structures of cellulase found in *Bacillus amyloliquefaciens* and various Bacillus species [24]. As a result, a semi-rational approach has emerged as a viable strategy for protein redesign. This understanding has resulted in significant advancements in catalytic activity, enzyme binding efficiency, and practical benefits for industries, agriculture, and medicine [25].

In this investigation, the strain of *Bacillus amyloliquefaciens* was isolated from rice peel and molecularly identified, revealing its potential for cellulase production. Our objective was to optimize the cultural conditions for maximization of cellulase production from *Bacillus amyloliquefaciens* using the methods of one-factor-at-a-time (OFAT) and BOX–Behnken Design (BBD). The data obtained from this research can provide insights into the specific conditions influencing cellulase yield for practical application. Furthermore, we cloned and heterologously expressed the cellulase-encoding gene, resulting in significant cellulase production. Moreover, our study highlights the value of in silico analyses of the cellulase encoded by the egl gene, which led to enhanced predictions for enzyme binding and catalytic activity. Cellulase production optimization was accomplished through the utilization of both the one-factor-at-a-time (OFAT) approach and the BOX–Behnken Design (BBD) method.

## Results

### Isolation, screening, selection and molecular identification of CMCase producing *bacteria*

**Fig. 1:**
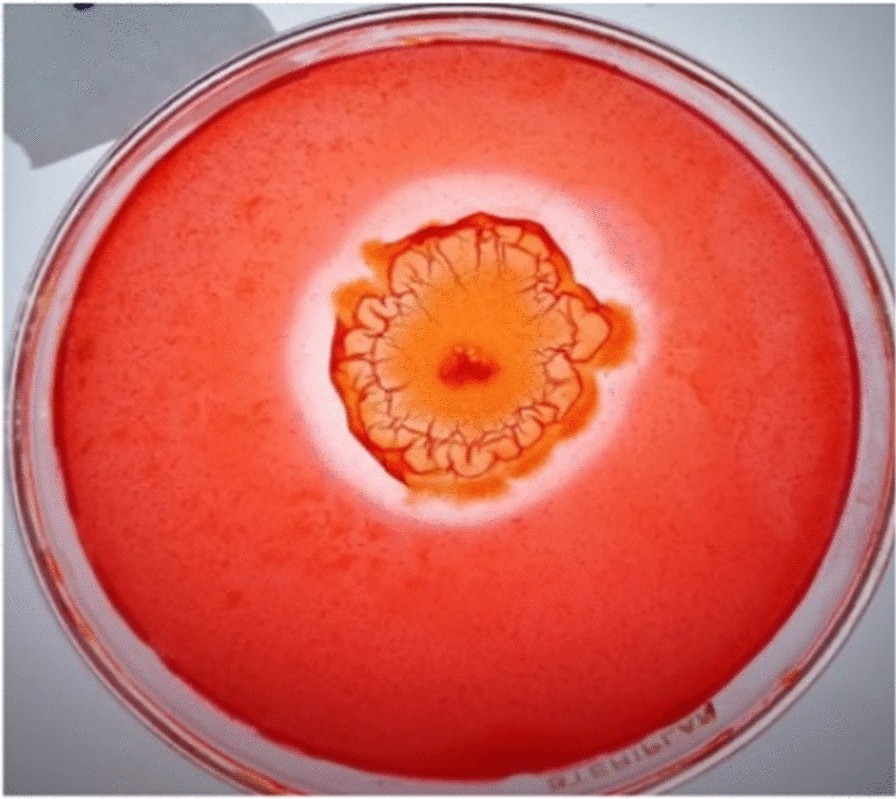
Zone of hydrolysis produced on nutrient agar plates supplemented with 1% (W/V) CMC by bacterial isolate elh1

**Fig. 2:**
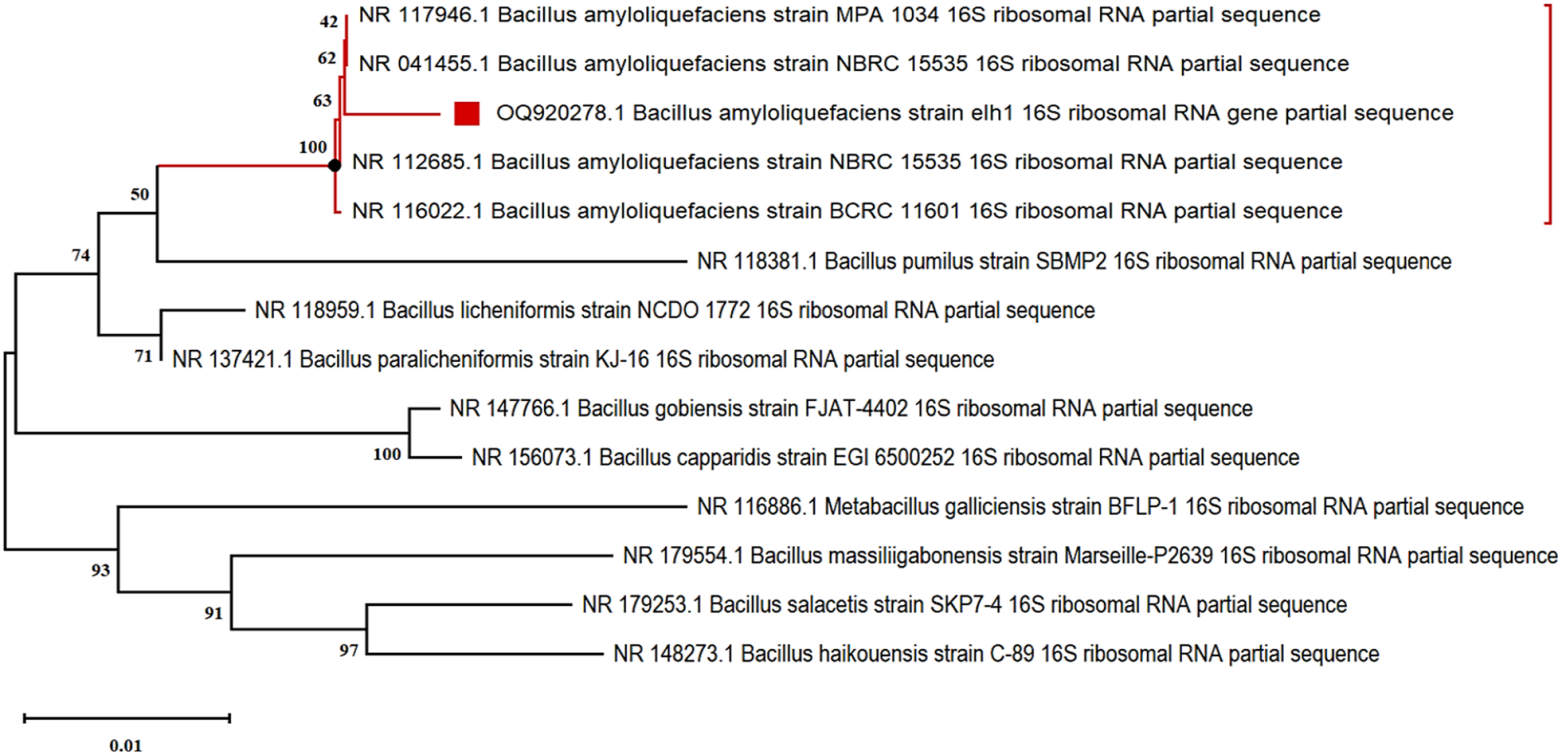
Phylogenetic Tree Reveals Evolutionary Relationships of Bacterial Strain elh1, indicated by a red square, within the Bacillus Genus

Among forty-seven bacterial isolates obtained from agriculture wastes suspensions, eleven strains, each with distinct morphology, tested positive in the zone of clearance test. The strain that exhibited the largest zone of clearance (Fig. 1) was chosen for further evaluation of enzyme production. The highest production of carboxymethyl cellulose (CMC) hydrolase (CMCase), 0.141 ± 0.006 U/ml, was reported by the bacterium that was originally isolated from rice peel and was designated as isolate elh1. Genotypic characterization based on nucleotide homology, a phylogenetic analysis of 16S rDNA sequence, revealed that the isolate elh1 has 99% similarity with *Bacillus amyloliquefaciens* strain NBRC 15535 (Sequence ID: NR041455)*.* Phylogenetic trees were created using the neighbor-joining (NJ) method, as shown in Fig. 2. The analysis of the phylogenetic tree confirmed that the bacterial strain elh1 is classified under the Bacillus genus. The elh1 strain was submitted to the GenBank database and assigned the accession number OR920278. Based on the phylogenetic analysis, the bacterial isolate elh1 was identified as *Bacillus amyloliquefaciens* strain elh1.

### Optimization of CMCase production conditions

#### One variable at a time (OVAT)

**Fig. 3:**
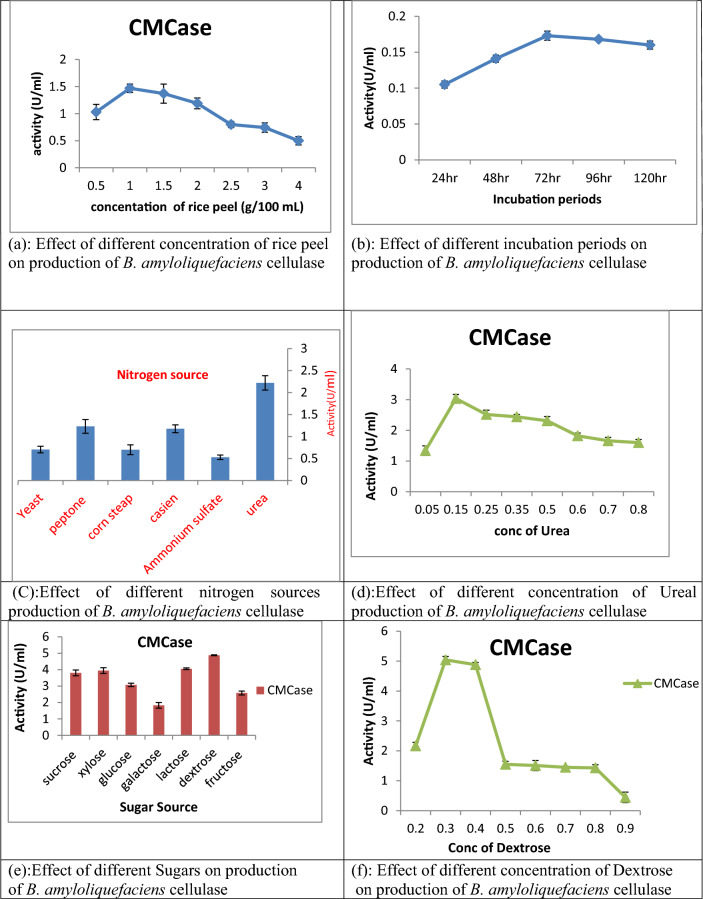
Effect of different factors on production of *B. amyloliquefaciens* cellulase

Choosing the appropriate strain is crucial for achieving effective enzyme production. However, to obtain the highest enzyme yield, it is necessary to meticulously optimize the production processes and culture conditions. This requires finely adjusting various parameters, inoculum size, incubation period, nitrogen source, sugars as a carbon source and substrate concentration, to maximize CMCase productivity. In this experiment, the rice peel—the efficient carbon source for CMCase production by *Bacillus amyloliquefaciens* strain elh1—was added in various concentrations; the maximum CMCase yield (0.141 ± 0.077 U/ml) was given at rice peel concentration of 1% (w/v) while the minimum production was detected at a concentration of 4% (Fig. 3a) after 48 h incubation. In terms of the temporal progression of CMCase production through submerged fermentation, Fig. 3b demonstrates that CMCase reached its peak production after 72 h of fermentation, with a yield of 0.173 ± 0.006 U/ml, and was reduced after 96 h. In this study, different nitrogen sources were applied to the production media; the component urea gave the highest CMCase yield (2.22 ± 0.162 U/ml) (Fig. 3c). Considering this data, urea was used in different concentrations; the productivity increased to 3.03 ± 0.131 U/ml at urea concentration of 0.15 g/L (Fig. 3d). Evaluating the efficacy of the various sugar supplements tested in this study in terms of their impact on CMCase production, dextrose was found to be the best sugar, where the yield reached 4.48 ± 0.028 U/ml (Fig. 3e) and increased to 5.04 ± 0.120 U/ml when dextrose concentration was 0.3% (w/v) (Fig. 3f).

### Statistical optimization of rice peel based‑medium for CMCASE production

#### The Plackett–Burman design (PBD)

**Fig. 4:**
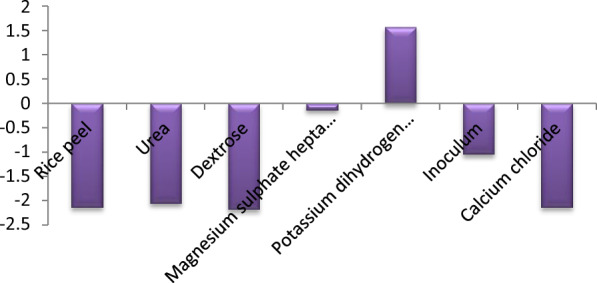
Main effects of independent variables on cellulase production according to the results of the PBD

For CMCase production, various process parameters were optimized and nutritional conditions were screened using placket Burman design of response surface methodology. The main nutritional components—seven variables—and eleven experiments were conducted for screening of various nutrients for CMCase production and results are mentioned in Table 1**.** The findings revealed the substantial impact of various factors on the fermentation process, as evidenced by the highest CMCase value (6.94 U/g) observed in trial 1, which included the following: rice peel (2 g/flask), Urea (0.05%), Dextrose (0.2 g/l00), MgSO_4_7H_2_O (0.25 g/l), KH_2_PO_4_ (4 g/l), CaCl_2_ (0.3 g/l) and inoculum (4 ml). In contrast, the lowest CMCase value (0.42 U/ml) was observed in trial 8, which consisted of rice peel (3 g/flask), Urea (0.25%), Dextrose (0.4 g/l00), MgSO_4_7H_2_O (0.75 g/l), KH_2_PO_4_ (4 g/l), CaCl_2_ (0.5 g/l), and inoculum (4 ml). The primary effects of the investigated parameters on CMCase production have been estimated and visually represented in Fig. 4. Analysis revealed that while the factors examined—rice peel, Urea, Dextrose, MgSO_4_ 7H_2_O, Inoculum, and CaCl_2_—exhibited negative effects, KH_2_PO_4_ showed a positive effect. The confidence level, P-effect, and t-test results of the statistical analysis of the Plackett–Burman Design (PBD) are detailed in Table 2. Consequently, due to the high significance level indicated by the P-value for the variables rice peel, Urea, and Dextrose, they were chosen for further optimization. The equation provided below represents a first-order model that elucidates the relationship between the seven components and CMCase activity:

$$ {\text{Y}}_{{{\text{activity}}}} = {1}0.{18}0{62} - {2}.{\text{52951 X}}_{{1}} - {1}0.{3}0{\text{84 X}}_{{2}} - {1}0.{9}0{\text{23 X}}_{{3}} - 0.{\text{28682 X}}_{{4}} + {1}.{\text{568245 X}}_{{5}} - 0.{\text{52281 X}}_{{6}} + {1}.{\text{946743 X}}_{{7}} $$

**Table 1:** Plackett–Berman experiment coded levels and real values

Trial	Rice peel	Urea	Dextrose	MgSO4 7H2O	KH2PO4	Inoculum	CaCL_2_	Activity (U/ml)
1	2(−)	0.05(−)	0.2(−)	0.25(−)	4(+)	4(+)	0.3(−)	6.94 ± 0.353
2	3(+)	0.05(−)	0.4(+)	0.25(−)	3(−)	2(−)	0.5(+)	2.10 ± 0.290
3	2(−)	0.25(+)	0.4(+)	0.25(−)	3(−)	4(+)	0.3(−)	1.48 ± 0.353
4	3(+)	0.25(+)	0.2(−)	0.25(−)	4( +)	2(−)	0.5(+)	4.13 ± 0.081
5	2(−)	0.05(−)	0.4(+)	0.75(+)	4( +)	2(−)	0.3(−)	6.01 ± 0.148
6	3(+)	0.05(−)	0.2(−)	0.75(+)	3(−)	4(+)	0.5(+)	3.44 ± 0.049
7	2(−)	0.25(+)	0.2(−)	0.75(+)	3(−)	2(−)	0.3(−)	4.22 ± 0.071
8	3(+)	0.25(+)	0.4(+)	0.75(+)	4(+)	4(+)	0.5(+)	0.42 ± 0.063
9	1(0)	0.15(0)	0.3(0)	0.5(0)	2(0)	3(0)	0.4(0)	5.03 ± 0.064
10	1(0)	0.15(0)	0.3(0)	0.5(0)	2(0)	3(0)	0.4(0)	5.03 ± 0.064
11	1(0)	0.15(0)	0.3(0)	0.5(0)	2(0)	3(0)	0.4(0)	5.03 ± 0.064

R-square, 0.993942; adjusted R-square, 0.979807. *P*-value: 0.002538

**Table 2:** A statistical analysis of the Plackett–Burman design shows coefficient values, effect, t- and P-values for each variable on the cellulase study

	Coefficients	Standard error	t stat	P-value
Intercept	10.18062	0.871588	11.68054	0.001348
Rice peel	−2.52951	0.235685	−10.7326	0.00173
Urea	−10.3084	0.993327	−10.3777	0.001909
Dextrose	−10.9023	0.993327	−10.9755	0.001619
MgSO_4_ 7H_2_O	−0.28682	0.397331	−0.72186	0.522558
KH_2_PO_4_	1.568245	0.198665	7.8939	0.004237
Inoculum	−0.52281	0.099333	−5.26321	0.013365
CaCL_2_	1.946743	1.541228	1.263111	0.295792

#### box–behnken design (BBD)

**Fig. 5:**
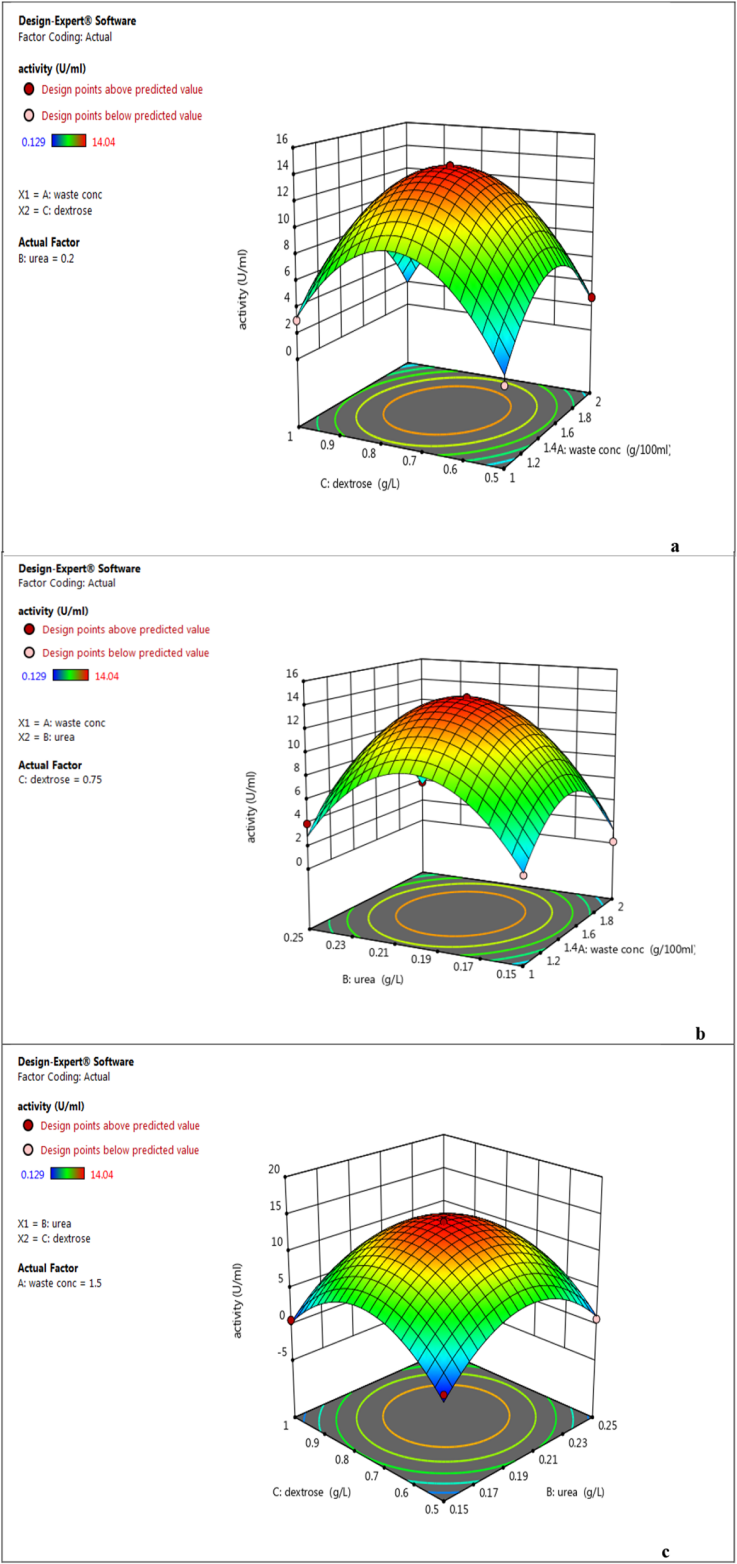
Three-dimensional response surface plots showing the effect of different variables on cellulase production: waste concentration, Dextrose (**a**); waste conc, Urea (**b**); Urea, Dextrose (**c**). Green, yellow, and red color showed low, medium, and high cellulase activity (U/mL), respectively

To optimize the concentrations of rice peel, Urea and Dextrose, Box–Behnken design of response surface methodology with three levels (−1, 0, and + 1) obtained through 17 experimental runs. The independent variables were assessed at five discrete levels within the experimental design. Table 3 displays the theoretical and observed increases in CMCase derived from the statistical analysis of the test factors. The maximum CMCase production (14.04 ± 0.424 U/ml) was observed using 1.5% rice peel, 0.2% Urea and 0.75% Dextrose (experimental run#1). The predicted CMCase production under these conditions were almost near to the observed value depicting the accuracy of the model. The analysis of variance for the quadratic regression model revealed a highly significant F-value of 74.47 (Table 4). The adjusted coefficient of determination, “Adj R-Squared,” was calculated to be 0.9764, resulting in a predictive R Square value of 0.8346, which is deemed very high according to the F-test (Tables 4 and 5). The R-squared value indicates the proportion of variability in observed response values that can be explained by the experimental factors and their interactions. The effects of interactions and variable responses were examined using the Box-Behnken design (Fig. 5).

**Table 3:** Examined concentration of the key variables and results of BBD experiment

Run	Factor 1 A:waste conc g/100 ml	Factor 2 B:urea g/L	Factor 3 C:dextrose g/L	Response 1 activity U/ml	Predicted value	Residual
1	1.5(0)	0.2(0)	0.75(0)	14.04 ± 0.424	14.04	0.0000
2	1(−)	0.25(+)	0.75(0)	3.92 ± 0.076	2.81	1.12
3	1.5(0)	0.15(−)	0.5(−)	0.414 ± 0.027	−0.5032	0.9178
4	2(+)	0.2(0)	1(+)	2.672 ± 0.126	1.93	0.7399
5	1.5(0)	0.2(0)	0.75(0)	14.04 ± 0.424	14.04	0.0000
6	1.5(0)	0.2(0)	0.75(0)	14.04 ± 0.423	14.04	0.0000
7	2(+)	0.2(0)	0.5(−)	2.723 ± 0.118	2.53	0.1979
8	1.5(0)	0.25(+)	1(+)	0.677 ± 0.066	1.59	−0.9179
9	2(+)	0.25(+)	0.75(0)	3.825 ± 0.202	3.65	0.1780
10	1(−)	0.2(0)	1(+)	2.989 ± 0.043	3.19	−0.1979
11	1.5(0)	0.2(0)	0.75(0)	14.04 ± 0.424	14.04	0.0000
12	1(−)	0.2(0)	0.5(−)	0.662 ± 0.033	1.40	−0.7399
13	1(−)	0.15(−)	0.75(0)	2.032 ± 0.154	2.21	−0.1780
14	1.5(0)	0.2(0)	0.75(0)	14.04 ± 0.424	14.04	0.0000
15	2(+)	0.15(−)	0.75(0)	0.129 ± 0.001	14.04	0.0000
16	1.5(0)	0.25(+)	0.5(−)	0.799 ± 0.014	2.81	1.12
17	1.5(0)	0.15(−)	1(+)	0.644 ± 0.031	−0.5032	0.9178

**Table 4:** ANOVA for the quadratic response surface model (RSM) from the cellulase production

Source	Sum of squares	df	Mean square	F-value	p-value (prob > F)
Model	545.14	9	60.57	74.47	< 0.0001*
A-waste conc	0.0086	1	0.0086	0.0106	0.9208
B-urea	4.52	1	4.52	5.55	0.0506
C-dextrose	0.7089	1	0.7089	0.8715	0.3816
AB	0.8095	1	0.8095	0.9951	0.3517
AC	1.41	1	1.41	1.74	0.2289
BC	0.0309	1	0.0309	0.0380	0.8510
A^2^	103.86	1	103.86	127.69	< 0.0001*
B^2^	183.11	1	183.11	225.11	< 0.0001*
C^2^	195.37	1	195.37	240.18	< 0.0001*
Residual	5.69	7	0.8134		
Lack of fit	5.69	3	1.90		
Pure error	0.0000	4	0.0000		
Std. Dev	0.9019		R^2^	0.9897	
Mean	5.39		Adjusted R^2^	0.9764	
C.V. %	16.72		Predicted R^2^	0.8346	
			Adeq Precision	21.0246	

^*^Significant variable at 95% confidence. R^2^: 0.9897, R^2^ Adj: 0.9764. Adequate precision ratio: 21.0246. DF, degree of freedom

**Table 5:** Coefficients in terms of coded factors

Factor	Coefficient estimate	df	Standard error	95% CI low	95% CI high	VIF
Intercept	14.04	1	0.4033	13.09	14.99	
A-waste conc	−0.0329	1	0.3189	−0.7869	0.7212	1.0000
B-urea	0.7513	1	0.3189	−0.0027	1.51	1.0000
C-dextrose	0.2977	1	0.3189	−0.4563	1.05	1.0000
AB	0.4499	1	0.4509	−0.6165	1.52	1.0000
AC	−0.5945	1	0.4509	−1.66	0.4719	1.0000
BC	−0.0879	1	0.4509	−1.15	0.9784	1.0000
A^2^	−4.97	1	0.4395	−6.01	−3.93	1.01
B^2^	−6.59	1	0.4395	−7.63	−5.56	1.01
C^2^	−6.81	1	0.4395	−7.85	−5.77	1.01

#### The model validation

**Fig. 6:**
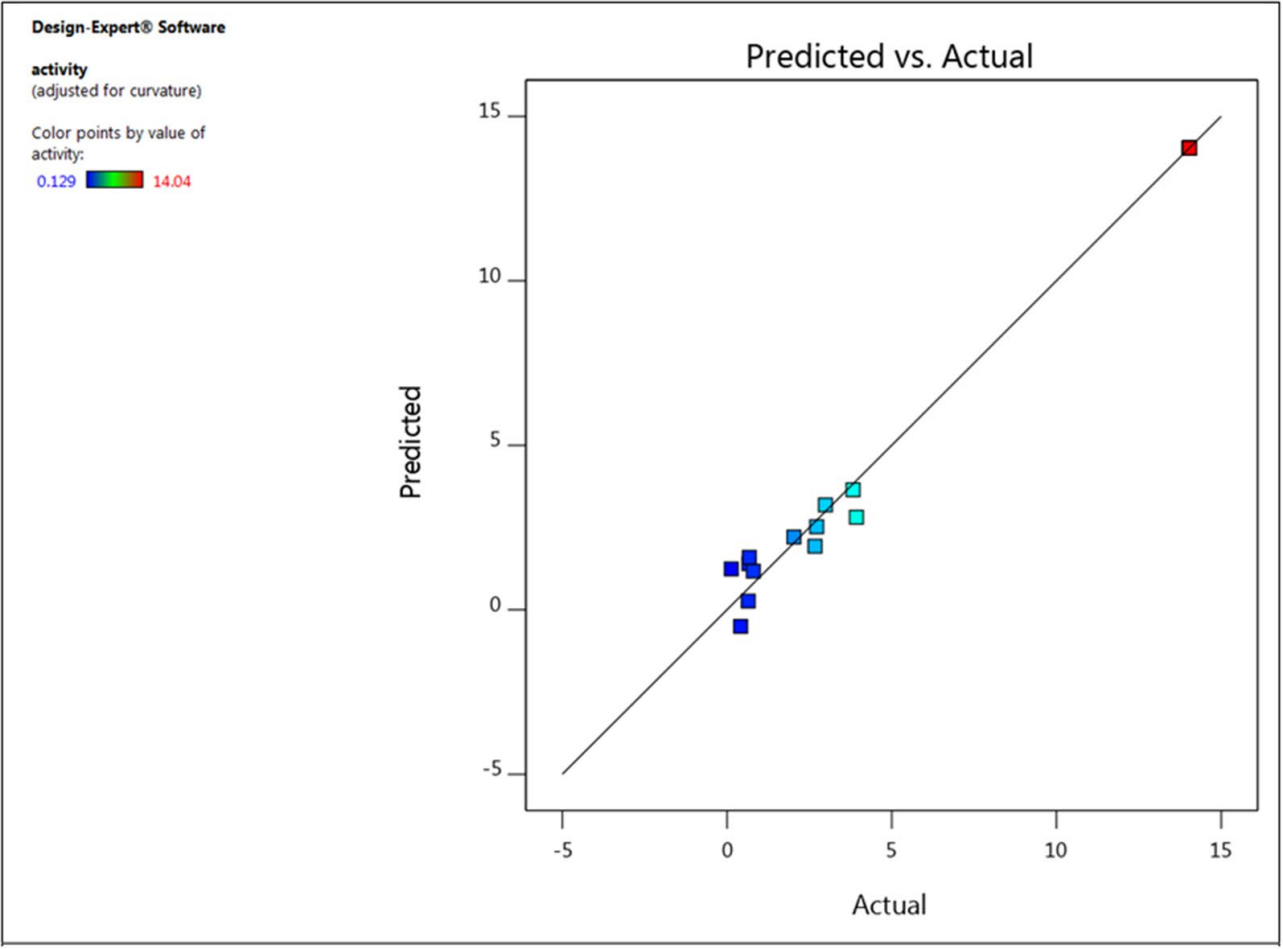
Residual plot of the observed-predicted values (residuals) versus the response (optimization process) of *B. amyloliquefaciens* CMCase

To verify the model's accuracy, a set of 17 random production combinations was employed to conduct experimental retests of CMCase production. The optimized conditions determined from the model projected a CMCase production of 14.04 U/ml. Impressively, the experimental value obtained precisely matched the predicted value, indicating the validity and reliability of the model. This close alignment between the experimental and predicted values is further supported by the data presented in Table 4. The Lack of Fit F-value of 1.34 suggests that the Lack of Fit is not significant compared to the pure error, with a 39.24% likelihood that a Lack of Fit F-value of this magnitude could occur due to noise. A non-significant Lack of Fit is desirable as it indicates a well-fitting model. The Predicted R2 of 0.7798 closely corresponds to the Adjusted R2 of 0.9063, with the difference being less than 0.2. The Adeq Precision, indicating the signal-to-noise ratio, demonstrates an adequate signal with a ratio of 14.967, suggesting that this model can effectively explore the design space. It is advisable to select the highest-order polynomial where the additional terms are significant and the model is not aliased. The Model F-value of 74.47 suggests the model's significance. With only a 0.01% likelihood of such a large F-value arising from noise, it underscores the model's robustness. P-values below 0.0500 signal significant model terms; in this instance, A^2^, B^2^, and C^2^ are notable. Conversely, values surpassing 0.1000 indicate insignificant model terms (Fig. 6).

### Amplifcation and cloning of egl gene from *Bacillus amyloliquefaciens strain* elh1

**Fig. 7:**
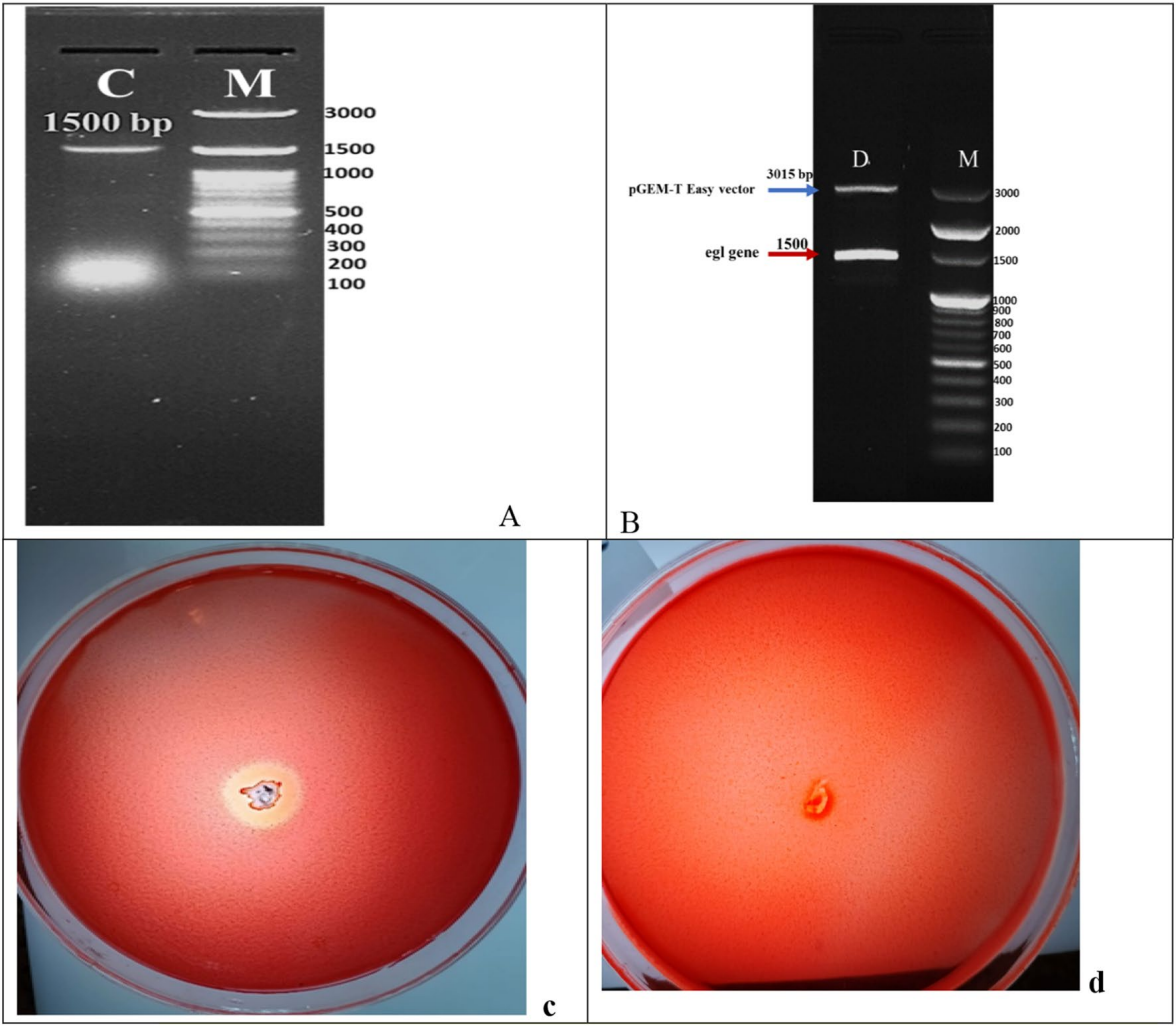
Validation of pGEM-egl Recombinant Construct Assembly through Multiple Techniques: **A** Colony PCR of Positive Transformants: The amplified fragment of the egl gene was visualized on an agarose gel, with the expected size of approximately 1500 base pairs (**C)**. The DNA ladder used for size reference was the 100 bp DNA ladder H3 RTU (GeneDirex, Inc.)(M). **B** Double Digestion of the Recombinant Construct: the egl gene fragment (indicated by a red arrow) and the linearized pGEM Teasy vector (indicated by a blue arrow). The DNA ladder used for size reference was the 100 bp DNA Ladder SolisFAST^®^(M). **C** Cellulase Activity Assay: The appearance of a clear zone of hydrolysis around the transformant colonies confirmed the expression and activity of the egl gene (**c**), absence of hydrolysis zone in case of non-transformant *E.coli* DH5α (**d**)

Using the genomic DNA of *Bacillus amyloliquefaciens* strain elh1 as a template, the particular primers created for the egl gene successfully amplified a DNA fragment of about 1500 base pairs. Using colony PCR, a gene fragment of roughly 1500 base pairs was amplified using the egl-F/egl-R primers, and the resulting construct was dubbed pGEM-egl. This confirmed the correct assembly of the construct (Fig. 7A). Subsequent double digestion assays confirmed the integrity of the pGEM-egl vector. The digestion yielded a fragment size of 3015 base pairs for the pGEM Teasy vector and approximately 1500 base pairs for the egl gene fragment (Fig. 7B). This dual confirmation through colony PCR and double digestion ensures the successful construction and proper assembly of the pGEM-egl vector, demonstrating the accurate incorporation of the egl gene fragment into the vector construct.

### CMCase heterologous expression in *E. coli* DH5α

The *E. coli* colonies containing the recombinant vector pGEM-egl were grown on nutrient agar plates supplemented with 1% carboxymethyl cellulose (CMC). Clear zones were observed around these cells, in contrast to the non-transformed *E. coli* cells (Fig. 7C, D), indicating the presence of cellulase activity. These colonies were then chosen for CMCase evaluation. The expression levels of the enzyme showed slight variation among the positive colonies tested, with the highest production reaching 22.3 ± 0.24 U/ml.

### DNA sequence and phylogenetic analysis of CMCase encoding gene (egl)

**Fig. 8:**
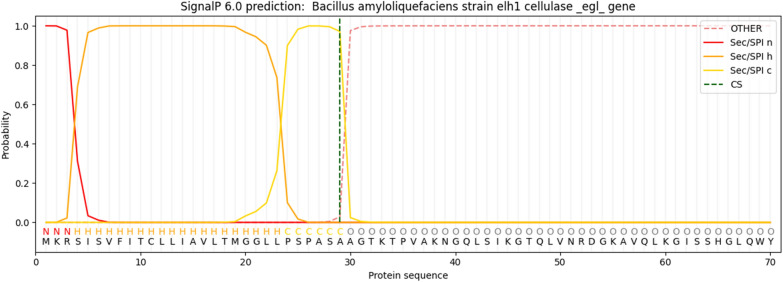
Analysis of cellulase signal peptide and pro-peptide using signalp version 6.0

**Fig. 9:**
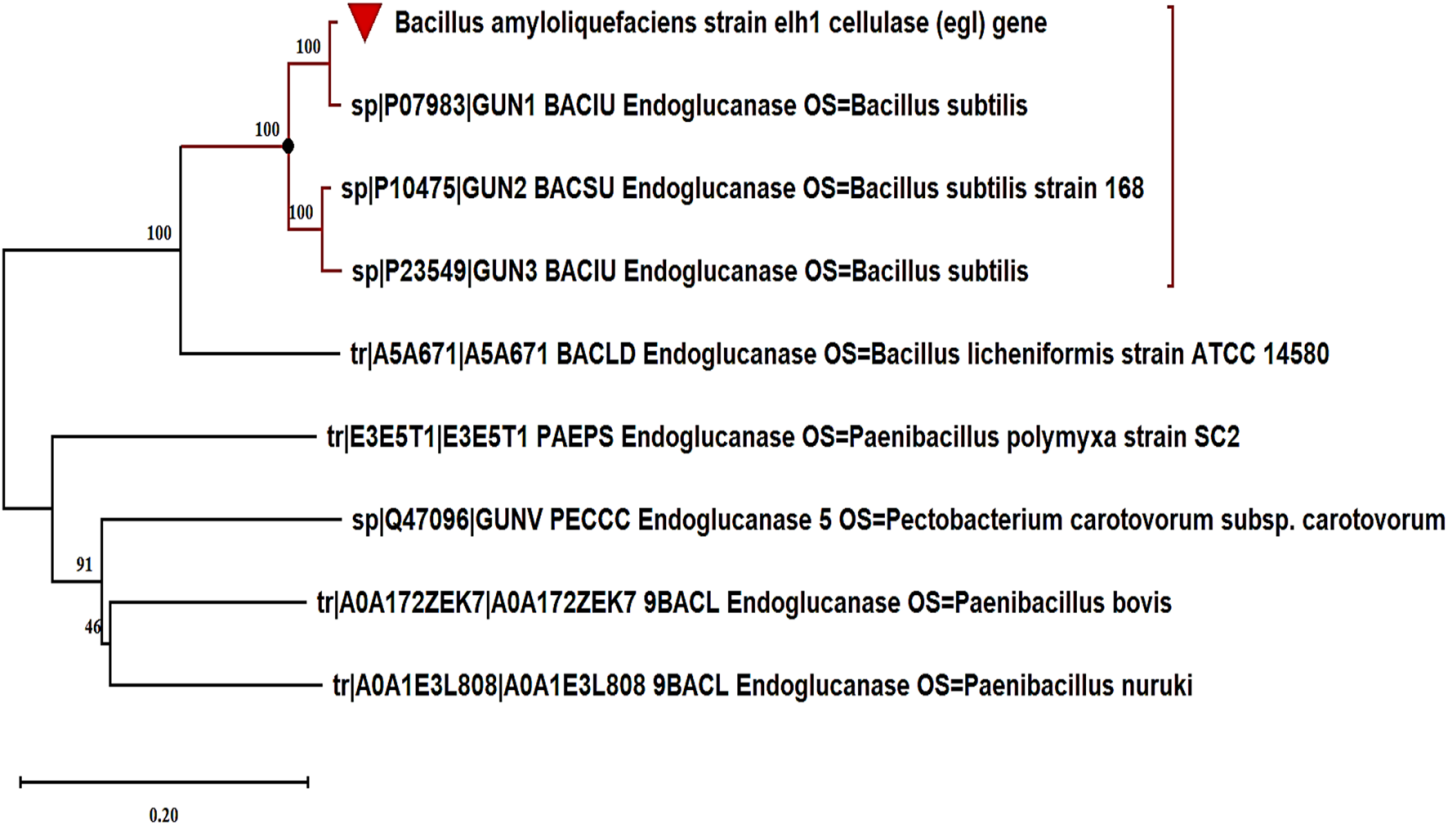
Phylogenetic tree of the egl inferred amino acid sequence (red triangle) and its nearest relatives, reconstructed in mega 11 through the neighbor-joining method from the uniprot database. bootstrap percentages are shown from 1000 replications

**Fig. 10:**
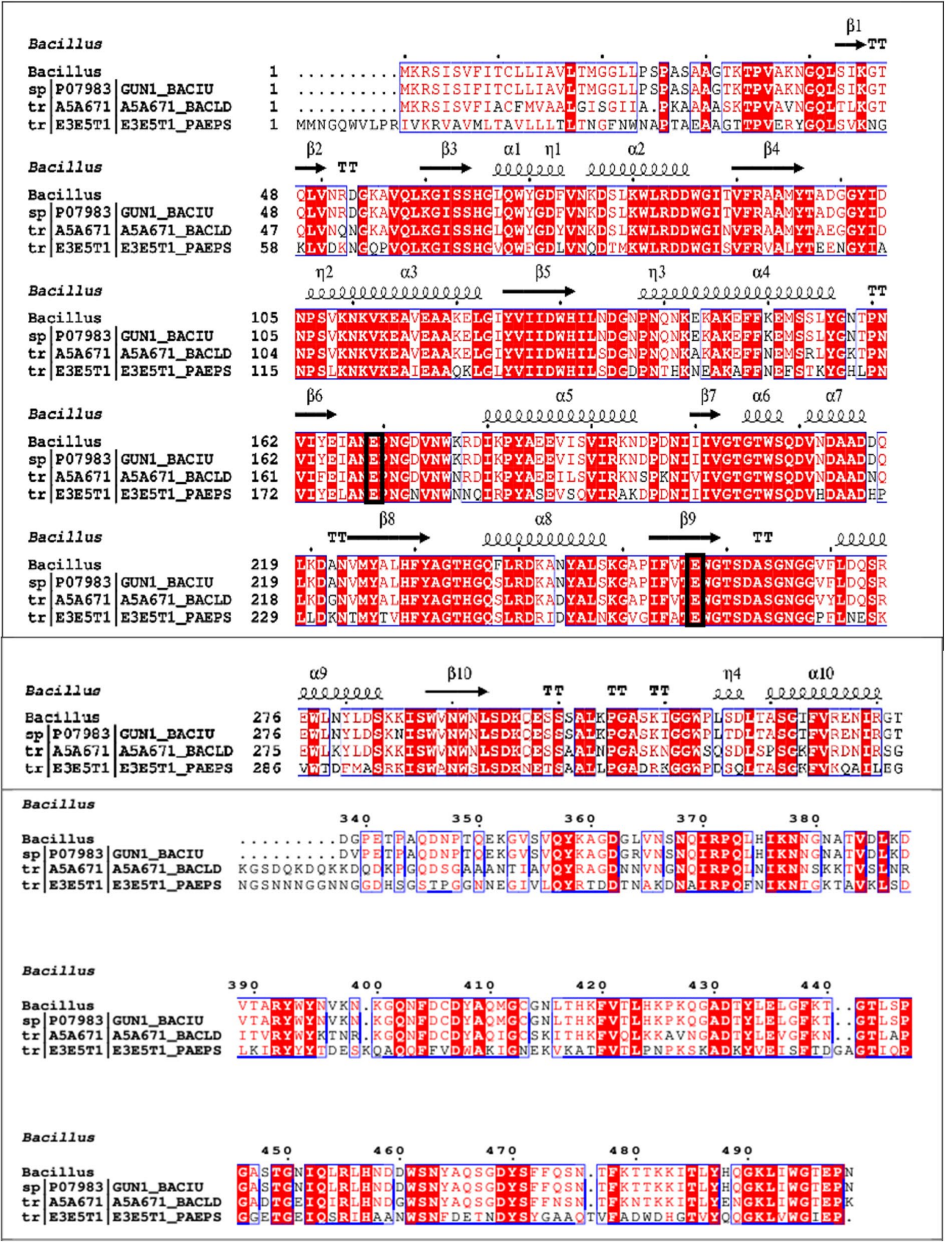
Multiple structure alignment of deduced amino acids sequence of egl gene and relative proteins retrieved from UniProt and rendering of secondary structure information regarding the prediction of α-helice and β-sheet regions. Residues invariable among sequences are typed in red on a white background; residues conserved within each group are displayed as white letters on a red background, and the residues representing active sites are shown in a black box. Secondary structure elements from known endoglucanase structure are indicated at the top of the alignment. TT letters represent strict beta turns

When comparing the predicted amino acid sequence of CMCase with homologous sequences in the UniProt database, a remarkable 98.8% similarity to the *Bacillus subtilis* endoglucanase entry P07983 was discovered. After submission to GenBank, the nucleotide sequence was assigned the entry number PP194445. This 1500 base pair open reading frame (ORF) encodes a pre-pro-enzyme consisting of 499 amino acids.The analysis indicated that the CMCase belongs to the glycosyl hydrolase family 5. SignalP analysis identified residues 1–29 as the signal peptide, responsible for the enzyme’s excretion (Fig. 8). The evolutionary relationship between the inferred amino acid sequence and the closest relatives from the UniProt database is shown in Fig. 9. The ESPript server was used to align the sequence of CMCase with its homologs, and the results, along with the secondary structure prediction, are shown in Fig. 10. The analysis of the CMCase model reveals a secondary structure consisting of 10 α-helices and 10 β-sheets. Additionally, two glutamic acid residues, Glu169 and Glu257, highlighted in black boxes, are identified as the active sites involved in the CMCase binding to the substrates.

### Homology modelling and structure validation of modelled CMCase

**Fig. 11:**
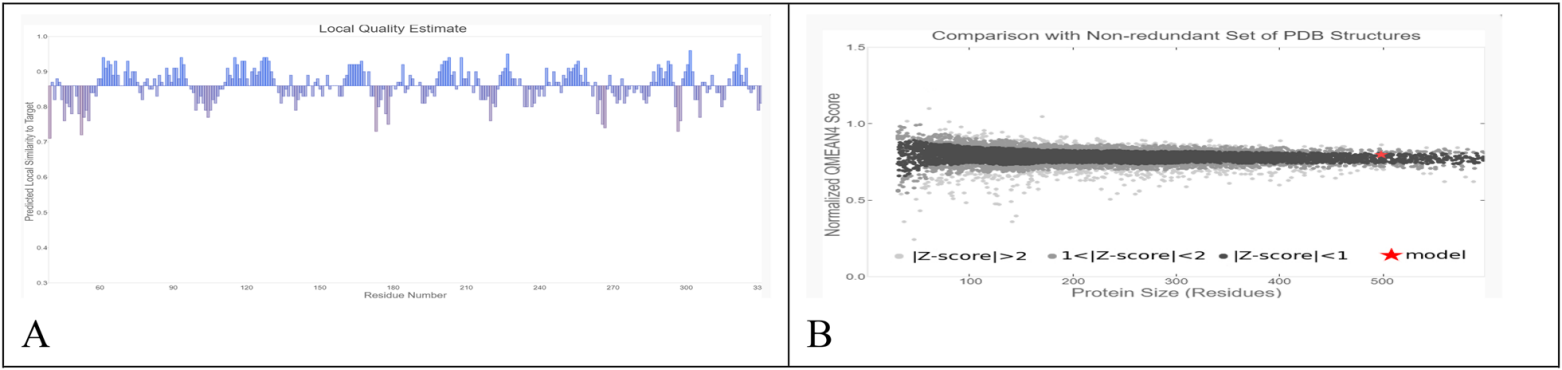
Validation of the modeled cellulase structure involves: **A** The evalation of local quality of the residues in the anticipated cellulase model; **B** the comparison of the predicted cellulase structure to a nonredundant set of PDB structures

**Fig. 12:**
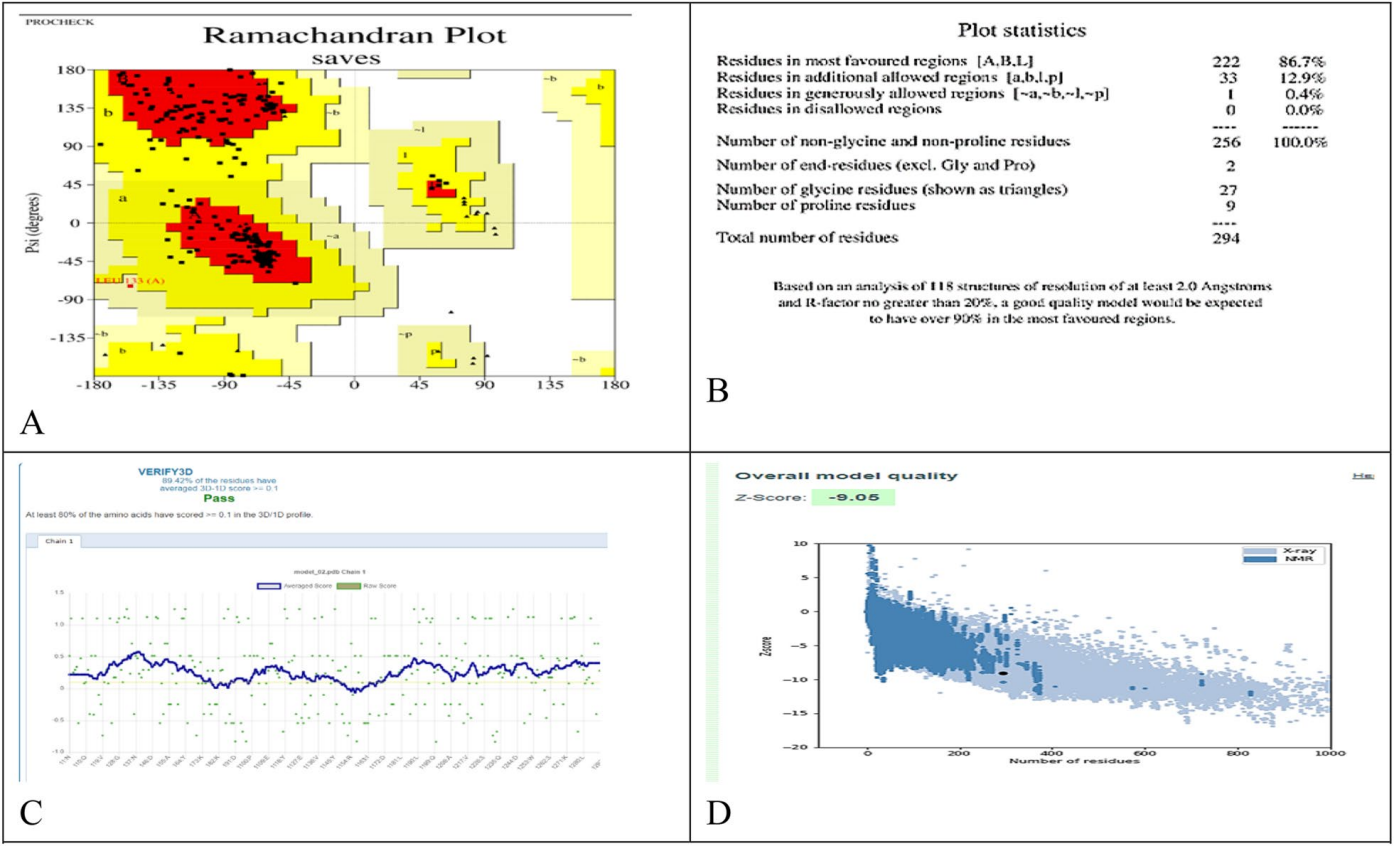
Estimated Model Assessment: **A** Ramachandran Plot Analysis; **B** Ramachandran Plot Statistics; **C** Verify3D Results; D ProSA-web Z-Score

Using SWISS-MODEL, a 3D model of CMCase was created; *Bacillus subtilis* 168 endo-1,4-beta-glucanase (PDB ID: 3PZT.1; resolution: 1.97 Å) was the best match. With a zero e-value, a GMQE value of 0.80, a QMEAN of 0.08, and a noteworthy 97.6% identity, the model demonstrates its high reliability and quality. The majority of residues, as shown in Fig. 11A, have values that are near to 1, which indicates good estimations of local quality. Low quality residues were defined as those having values less than 0.6. To further support its reliability, the modeled CMCase structure aligns perfectly with other protein structures in the PDB (Fig. 11B). The Ramachandran plot (Fig. 12A) and accompanying statistics (Fig. 12B) reveal that 86.7% of the modeled cellulase residues are in the most favored regions, 12.9% are in additional allowed regions, and 0.4% are in generously allowed regions, confirming the model’s high quality. The model’s validity was confirmed by the Verify3D plot (Fig. 12C), which showed a PASS for structural validation with a mean 3D-1D score of > 0.1 for 89.4% of residues. The remarkable quality of the anticipated model was indicated by the Z-score of −9.05 obtained from ProSA-web analysis (Fig. 12D). The 3D model's correct positioning of the protein backbone dihedral angles phi (φ) and psi (ψ) is verified by this investigation. Additionally, the ERRAT Complete Overall Quality Factor, which plots error function values against the location of a 9-residue sliding window, was 96.8085. This factor evaluates the statistics of non-bonded interactions among different types of atoms.

### Alignment of the CMCase model and template (3PZT) structure

**Fig. 13:**
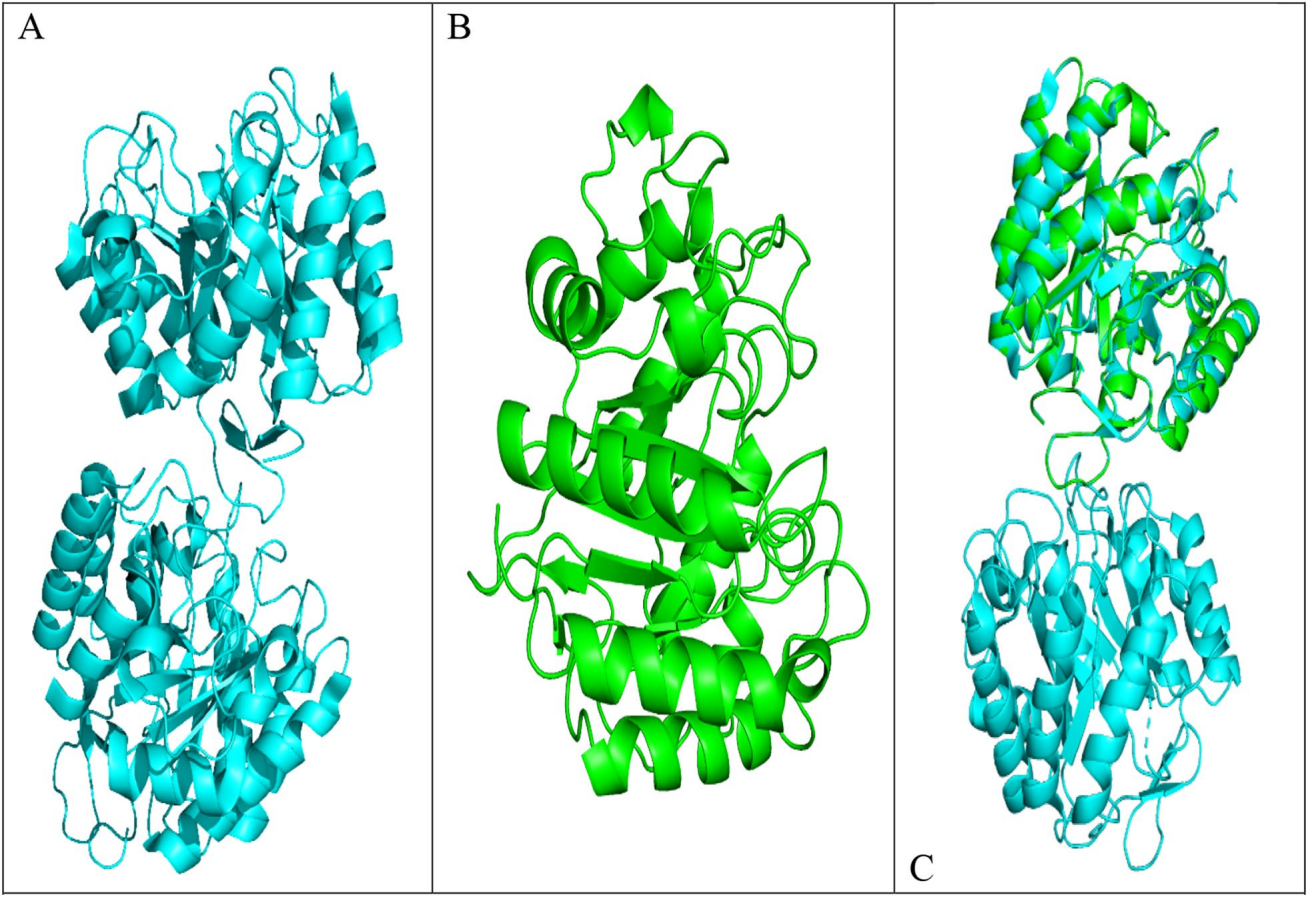
**A** The three-dimensional structures of 3PZT (template model); **B **the cellulase homology model; **C** Structure Alignment Between the Cellulase Target Protein and the Template Protein (3PZT)

Figure 13A, B show the 3D representations of CMCase and its template (3PZT), respectively. Alignment calculated using PyMOL Molecular Viewer revealed an RMSD value of 0.062 (285 to 285 atoms), indicating a close structural similarity (Fig. 13C). In the figures, the template structure is represented by cyan helices, while the protein homology model is represented by green helices. The template is homodimers and has two identical chains, A and B. The alignment of chain A in the template (3PZT) with the homology model demonstrates the high quality of the model.

### Investigations of docking and molecular interactions

**Fig. 14:**
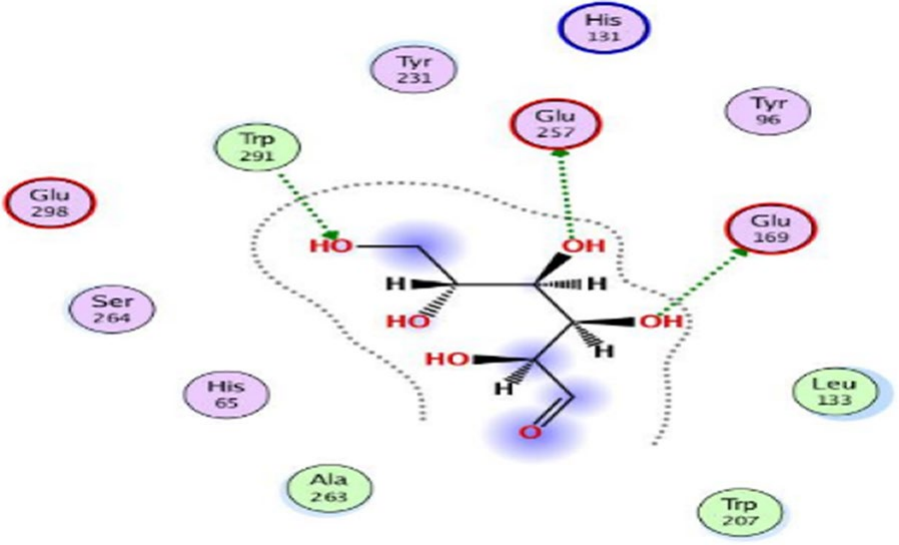
Molecular docking interaction of carboxymethyl cellulose with cellulase from *Bacillus amyloliquefaciens* Strain elh1

**Fig. 15:**
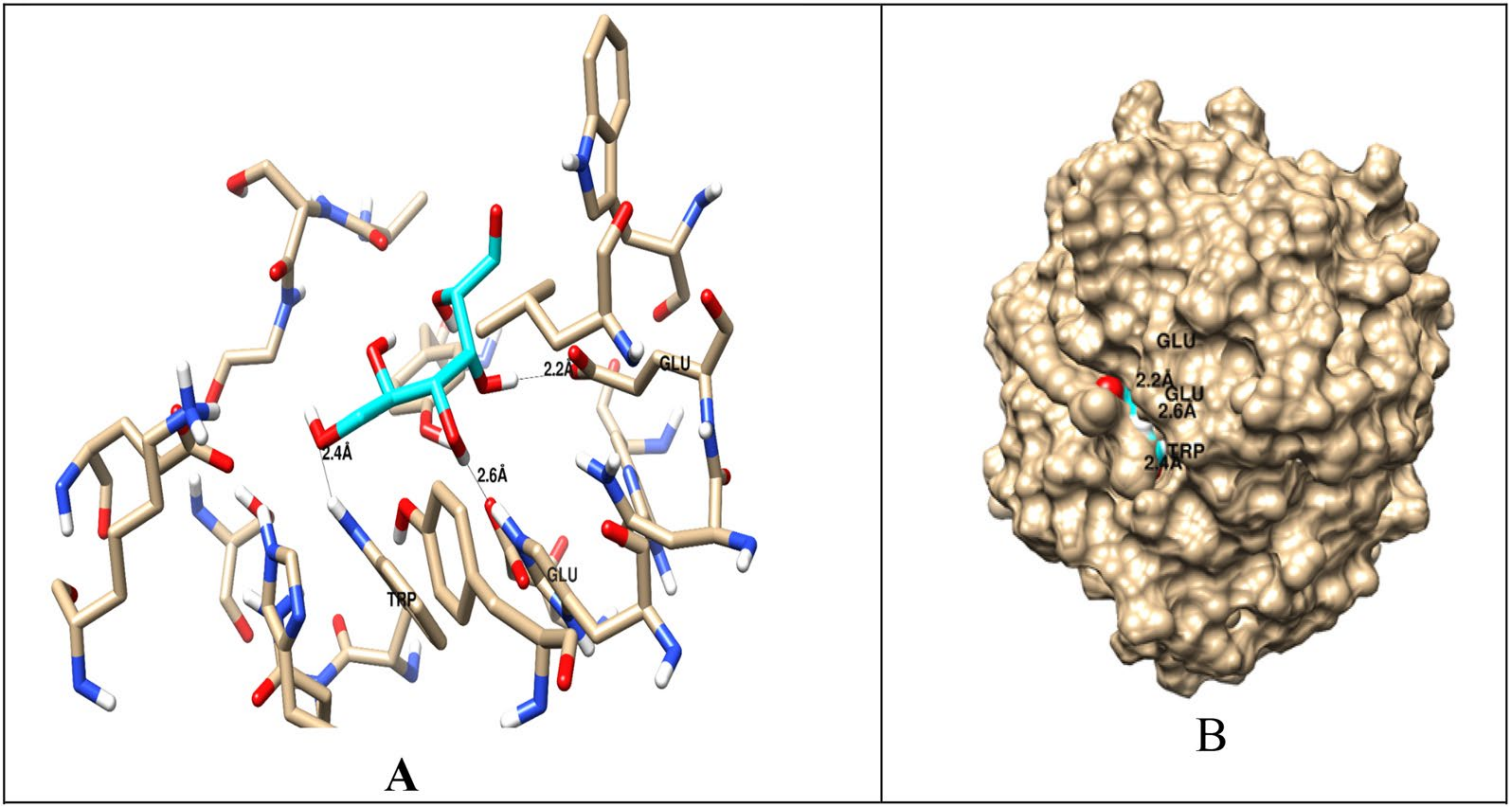
**A** Chimera software was used to visualise the binding disposition and receptor-ligand interactions of carboxymethyl cellulose (the carbon skeleton is displayed in cyan) at the cellulase binding site. **B** 3D Complex Interaction

As demonstrated by Fig. 14, the two hydroxyl groups on the ligand, carboxymethyl cellulose, create two hydrogen bonds with the CMCase protein within the binding pocket. The glutamic acid residues Glu169 and Glu257 are involved in these bonds, with corresponding distances of 2.2 and 2.6 Å. Glu169 and Glu257’s oxygen atoms accept hydrogen bonds from both hydrogen bonds, acting as hydrogen bond donors (HBD), resulting in an interaction affinity score of −5.71 kcal/mol. Additionally, a hydrogen bond with a distance of 2.4 Å is formed with the residue Trp219 (tryptophan). Visualization using Chimera revealed three hydrogen bonds and clearly showed the 3D structural interactions (Fig. 15). The angles of the hydrogen bonds were measured at 170.9º, indicating that they are reliable and effective.

## Discussion

This research comprised two main investigations: firstly, the production and optimization of a cellulase derived from *Bacillus amyloliquefaciens* strain elh1, in the presence of rice straw as a substrate; secondly, the cloning of egl gene, the characterization and in silico analysis of the protein. The accumulation of agricultural waste presents a significant environmental challenge as it contributes to pollution and requires a large amount of land for disposal. Addressing this issue can be costly, so it is crucial to explore sustainable solutions [2]. These wastes are primarily broken down by specific microorganisms that naturally reside in various environments. These microorganisms produce enzymes that break down lignocellulosic materials into simpler sugars [26]. Utilizing these waste materials and transforming them into valuable products offers economic benefits and reduces environmental impact. This represents a shift away from traditional harmful chemical treatments towards safe and sustainable alternatives. Cellulose-degrading microbes can be found in diverse habitats such as animal waste, gastrointestinal tracts, soil, and aquatic ecosystems. However, current population densities of these microbes are not sufficient to meet industrial demands. The predominant microorganisms of significance include bacteria and fungi, with bacteria being particularly favored over fungi due to their adaptability to industrial environments. Ongoing research efforts are focused on exploring different ecological niches to isolate bacterial strains that exhibit promising cellulolytic activity.

In this study, a robust bacterial strain called *Bacillus amyloliquefaciens* elh1was isolated. This strain was chosen for its significant cellulase production when cultured on a 1% carboxymethyl cellulose (CMC) at 37 °C for 48 h (0.141 ± 0.077 U/ml). Similar studies have also demonstrated the effectiveness of these approaches in utilizing bacterial strains for cellulase production. In a similar study, Islam and Roy [27] discovered that the bacterial species *Paenibacillus* sp. exhibited considerable promise for achieving maximum cellulase production (0.9 µmol ml^−1^ min^−1^) at pH 7.0 following a 24 h incubation period at 40 °C. This observation was made in a medium comprising 1.0% CMC. In a study conducted by Fouda et al. [28], it was revealed that the dominant cellulase activity of *Bacillus amyloliquefaciens* M7 was measured at 11.6 ± 0.4 U/ml. In another investigation by Singh et al. [14], the CMCase activity observed in the cell-free supernatant of *B. amyloliquefaciens* SS35 ranged from 0.132 to 0.528 U/ml. Additionally, Ahmad et al. [29] isolated the bacterial strain *Aneurinibacillus aneurinilyticus* from urban Himalayan freshwater. This strain exhibited cellulolytic activity on 0.5% CMC agar and showed a zone of hydrolysis. The utilization of agro-wastes as substrates for cellulase production offers a comprehensive approach with dual benefits. Firstly, it helps to eliminate accumulated agro-wastes. Secondly, it facilitates the production of cellulase from a cost-effective source. In this investigation, rice straw was the best substrate for *Bacillus amyloliquefaciens* strain elh1 to produce cellulase enzyme. The study by Pham et al.[1] investigated the ability of *Bacillus* sp. to produce cellulase (140 U/ml) from coconut-mesocarp. Also, Bala et al. [5] exploited the cheap agro-waste, sugarcane bagasse as a substrate for cellulase enzyme production using *Bacillus licheniformis* MTCC 429. In this study, rice straw is identified as the optimal substrate for *Bacillus amyloliquefaciens* strain elh1 in the production of cellulase enzymes. The total quantity of cellulase is significantly influenced by nutritional and physical culture conditions [30]. Numerous researchers have explored cellulase yield in various media and production environments [29–31]. Initially, cellulase production in bacterial isolates was conducted at 37 °C and a pH of 7, consistent with findings from multiple published sources [20, 31]. In this investigation, we conducted an optimization of production medium supplements, focusing on carbon sources (sugars), organic nitrogen, incubation durations, and substrate concentration (rice straw) using a one-factor analysis approach. Among the carbon sources assessed, dextrose demonstrated the highest production yield compared to the other carbon sources examined in this study. These findings diverge from previous research, which identified glucose as the optimal carbon source for cellulase production [29, 32]. Another crucial determinant impacting cellulase activity involves the incorporation of supplementary organic and inorganic nitrogen sources into the production media. The greatest cellulase activity (30.9 ± 0.1 Uml^−1^) was noted when urea was employed as the nitrogen source, while utilization of alternative nitrogen sources resulted in a reduction in enzyme activity. However, Singh et al. [18] proposed yeast extract and peptone as the most effective organic nitrogen sources for cellulase production in *Bacillus amyloliquefaciens* SS35, whereas Fouda et al. [28] suggested peptone as the preferable choice over yeast extract for optimizing cellulase production in *Bacillus amyloliquefaciens* M7. Upon examining the impact of the incubation period, we observed that cellulase production commenced 24 h after incubation and experienced a significant increase, reaching its peak after 72 h. At the 72-h time point, the maximum cellulase production of 54.3 ± 0.1 Uml^−1^ was attained, after which it gradually declined. This decline can be elucidated by the bacterial strain’s progression through different growth phases. Initially, during the initial 24 h period, the bacterial strain enters a lag phase, wherein it adapts to the new environmental conditions and metabolizes the available nutrients. Subsequently, a phase of exponential growth follows, characterized by a rapid proliferation of bacterial strains. However, after reaching an optimal incubation period, cellulase activity diminishes due to a decline in the metabolic activity of bacterial species, possibly due to nutrient depletion or the accumulation of detrimental metabolites in the growth medium. Our findings align with the results reported by Ye et al. [19] but differ from those of Fouda et al. [28]. They concluded that the optimal incubation period for maximizing cellulase production is 24 h. The concentration of enzyme–substrate within fermentative media, such as rice peel, plays a crucial role in augmenting enzymatic activity. Employing the *B. amyloliquefaciens* strain elh1, the highest recorded cellulase activity (64.7 ± 0.3 Uml^−1^) was attained at a concentration of 1 g/L of rice peel. Beyond this threshold, cellulase activity gradually decreased due to substrate inhibition [33]. Inadequate availability of rice peel at lower concentrations (0.5 gL^−1^) resulted in reduced cellulase activity due to insufficient substrate for the enzymes to act upon. Conversely, substrate inhibition was observed at higher concentrations of rice peel. Increased substrate concentrations led to the accumulation of end products or intermediate metabolites, which hindered cellulase enzymes by disrupting active sites or catalytic activity, consequently diminishing enzyme efficacy. Hence, optimizing the equilibrium between enzyme activity and substrate concentration is imperative. Deviating from the optimal concentration range, whether lower or higher, negatively impacts enzyme activity either due to substrate insufficiency or substrate inhibition. It is important to note that when the concentration of rice peel increases, it absorbs the available liquid. This absorption then mitigates the agitation required for optimal aeration conditions. As a result, these conditions become unsuitable for ideal bacterial growth and consequently reduce cellulase production. The impact of inorganic salts on CMCase production is surprisingly minimal. As indicated by the negative Plackett–Burman model coefficient, all the factors exhibited negative effect on CMCase production except the potassium phosphate, which posed positive effect. It can be explained the well-known roles of potassium phosphate in cell growth-enhancing buffer solutions [32, 34, 35]

The detection of significant activity on carboxymethyl cellulose (CMC) suggests that the CMCase produced by *Bacillus amyloliquefaciens* strain elh1 may meet the criteria outlined by Coughlan and Mayer [36] for classification as an endoglucanase. Therefore, our investigation indicates that the enzyme produced by *Bacillus amyloliquefaciens* strain elh1 is likely functioning as an endo-β-1,4-glucanase, based on its specificity towards the substrate. The gene responsible for encoding an endo-β-1,4-glucanase was screened and characterized. The designed primers, egl-F and egl-R successfully amplified a 1500 base pairs fragment. This fragment was then cloned into the pGEM Teasy vector. The amplified fragment corresponds to the egl gene found in *Bacillus amyloliquefaciens* strain elh. It was aligned with sequences in GenBank and translated into deduced amino acids to validate the sequence. In silico analysis confirmed that the amplified fragment corresponds to the egl gene responsible for CMCase production. Our findings are consistent with the results reported by Sun et al. [37]. In their study, Sun and his colleagues successfully isolated a gene that is 1500 base pairs long and encodes a specific type of cellulase found in *B. amyloliquefaciens* S1. Thakkar and Saraf [38] identified a gene responsible for encoding cellulase, composed of 1300 base pairs in length. Subsequently, they conducted cloning of the gene, that was cloned into the pCR4-TOPOR vector. Moreover, cellulase-encoding genes of 1500 base pairs in length were also discovered and sequenced in various Bacillus species, such as *B. subtilis* (natto strain) [39] and *Bacillus subtilis* IARI-SP-1 [40]. The use of heterologous expression is a valuable strategy for enhancing cellulase production. Although primarily a cloning vector, the pGEM-Teasy vector effectively facilitates heterologous gene expression, particularly when the gene product is non-toxic to the host organism, *E. coli* DH5α. Numerous studies have employed the pGEM-Teasy vector for both cloning and heterologous gene expression. For example, Abdel-Salam et al. [41] and their colleagues successfully cloned and expressed the avicelase Gene from *Bacillus subtilis* subsp. subtilis 168 in *E. coli* DH5α using the pGEM-Teasy vector. Similarly, from *Rhodotorula mucilaginosa*, Abd El-Aziz et al. [42] proficiently cloned and expressed the gene that encodes Endo-polygalacturonase into *E. coli* DH5α via the pGEM-Teasy vector. Analysis of the inferred amino acid sequence of cellulase indicated its categorization within the Glycoside hydrolase family 5 (GH5) subfamily 1 (EC 3.2.1.4). Within GH5, Various conserved residues of amino acids were identified, namely histidine (H), asparagine (N), glycine (G), arginine (R), tyrosine (Y), glutamic acid (E), and tryptophan (W). These residues play a crucial task in the catalytic machinery, particularly glycine, arginine, and tryptophan, facilitating substrate binding and influencing hydrolytic activities [43]. Among these residues, two glutamic acids (E) were the most significant in the GH5 family. These residues of glutamic acid serve as proton donors and nucleophiles, respectively, thereby playing a pivotal role in the catalytic activity [8]. In our investigations the glutamic acid residues stand for Glu 169 and Glu 257.

Computational modelling and docking investigations are useful tools for exploring the relationship between bacterial cellulases and CMC. Investigating the structure–function connection and substrate-protein interactions of proteins is important, despite their limitations in comparison to experimental methods, especially given their affordability [44]. Web-based servers validated the 3D structural model of cellulase by employing evaluations and quality assessments through QMEAN4, Z-score, Ramachandran plot analyses and ERRAT. The QMEAN score helps us to understand the geometric aspects of protein structures and the arrangement of variable residues. A higher QMEAN4 score indicates a better structure, while negative scores indicate instability [46]. QMEAN4 predicts the overall quality of model structures by combining four descriptors: local geometry, distance-dependent interaction, ensure consistency between the predicted secondary structure and solvent accessibility, as well as solvation potential. In this study, the QMEAN4 score for the 3D structure of cellulase was 0.08, indicating proper folding into a compact three-dimensional entity. Notably, desirable QMEAN scores ranged from 0 to 1 [45]. Validation of the 3D structures was further confirmed through crystallography, as represented by the ERRAT values that are associated with structural resolution, assessing protein structures based on the distances between pairs of atoms. Higher 3D structure resolutions typically yield rates of around 95% or higher, while lower resolutions indicate an average overall quality factor of approximately 91% [46]. In this study, interestingly, the cellulase structure exhibited an overall quality factor, as indicated by the ERRAT value of 94.96%, suggesting satisfactory structural resolution. Furthermore, a Ramachandran plot was established to visualize the position of each amino acid residue. As a result, 86.7% of the residues were located in the most favored regions. According to this resulted percentage, the constructed model is considered to be of good quality [47]. Molecular docking analysis revealed that carboxymethyl cellulase formed favorable interactions with *Bacillus amyloliquefaciens* strain elh1-cellulase within the active site of the enzyme. Among the amino acids involved, two glutamic acid residues were identified. Specifically, Glu 169 acts as a proton donor, facilitating the protonation of the glycosidic bond and subsequent bond fission. Additionally, Glu 257 acted as a nucleophile, aiding the reaction by stabilizing the resulting carbonium ion intermediate. These residues form hydrogen bonds with the substrate, carboxymethyl cellulase, within the active site.

Two main criteria were used to validate the docking protocol. These were the binding score and root mean square deviation (RMSD). The docking protocol involved the cellulase substrate-binding site and CMC ligand. With an RMSD of 1.5 Å and a significant interaction affinity score of −5.71 kcal/mol, the chosen pose was found to be reliable. It should be noted that an RMSD below 2.0 Å indicates a favorable docking solution [48], along with the best scoring energy. Several other poses also met these criteria. To ensure accuracy in the selection process, it is highly advisable to select the best docking solution based on other structural considerations indicated for related ligands, in addition to the scoring function [49]. These docking orientations are consistent with previous research conducted by Maryanty et al. [50] who performed molecular docking of cellulose with cellulase as a ligand and identified the active site composed of Glu169, Glu257, and Trp207. The discrepancy lies in the amino acid tryptophan, where our results identified the tryptophan site as 219. Moreover, data from the web-based server https://www.uniprot.org/uniprotkb/P07983/entry verified the representation of the active site by the Glu169 and Glu257 residues. Additionally, these findings support previous studies by Santos et al. [52] who also identified Glu169 and Glu257 as critical residues involved in interactions with carboxymethyl cellulose (CMC).

## Conclusion

In this investigation, we conducted the production of the cellulase enzyme using the bacterial strain *Bacillus amyloliquefaciens* strain elh1. We achieved optimal cellulase production from *B. amyloliquefaciens* after a fermentation period of 72 h. We found urea was the most suitable nitrogen source, while dextrose was the optimal sugar. This resulted in a production increase to 5.04 ± 0.120 U/ml. By employing statistical optimization through Response Surface Methodology (RSM), we reached a peak cellulase activity of 14.04 ± 0.42 U/ml.

Furthermore, we successfully cloned and expressed the cellulase encoding gene, egl, in *E. coli* DH5α. The transformed cells exhibited a cellulase activity of 22.3 ± 0.24 U/ml. In silico analysis of the protein sequence provided insight into the physicochemical properties of the cellulase produced by *Bacillus amyloliquefaciens* strain elh1. Molecular docking helped elucidate the amino acids within the active sites that are involved in substrate binding, particularly carboxymethyl cellulose. Our future research plans to modify these specific amino acids through site-directed mutation to enhance cellulase functionality.

## Materials and methods

### Bacterial strains, growth media, and chemicals

The endophytic bacterial strain elh1 was isolated from rice straw. Non-transformed and transformed *E. coli* DH5α cells were cultured at 37 °C in Luria–Bertani (LB) without and with the appropriate antibiotics (ampicillin 50 μg ml^−1^), respectively. Kits for GeneJET Genomic DNA Purification, GeneJET Plasmid Miniprep, andT4 DNA Ligase, were purchased from Thermo Scientific^™^ (Waltham, Massachusetts, United States). Primers were manufactured by Macrogen Inc. (Amsterdam, the Netherlands). Takara Bio was the source of the EmeraldAmp^®^ GT PCR 2 × master mix (Shiga, Japan). Promega Co. provided the pGEM-T easy-cloning vector (Madison, WI, USA).

### Primary screening and selection of the cellulase producing bacterial isolates

Nine different agricultural wastes were chosen for the purpose of isolation of potential cellulase producing bacteria: rice straw (RS), palm kernel (PK), wheat bran (WB), saw dust (SD), rice peel (RP), corn stalks (CS), pomegranate peel (PP), olive kernel (OK) and garlic peel (GP). After grinding of these dry wastes, one gram of powder for each was dissolved in 50 ml sterile saline solution under aseptic conditions in 250 ml conical flask, which then were permitted to shake at 150 rpm for 1 h. The suspensions were serially diluted until the concentration of 10^−9^; 100 µL from each dilution were plated onto the nutrient agar medium and subjected to incubation at a temperature of 37 °C until appearance of bacterial colonies. Colonies displaying distinct morphologies were isolated, purified, and preserved on nutrient agar slants at 4 °C; this procedure was executed following the methodology described by Waghmare et al. [52]. The cellulolytic/CMCase activity of the bacterial isolates was assessed on nutrient agar plates supplemented with 1% (W/V) carboxymethyl cellulose (CMC) following the method described by Vu et al. [53]. Briefly, the plates underwent incubation at 37 °C for 48 h, followed by flooding with a 0.1% Congo red solution and subsequently treated with 1 M NaCl. The presence of a transparent zone surrounding the bacterial growth indicated the hydrolysis of CMC. The colony exhibiting the most extensive clearance zone was chosen for molecular characterization through 16S rDNA sequencing.

### Production and activity assay of extracellular CMCase enzyme

A single colony of the bacterium showing the largest zone of clearance was first cultured on nutrient broth overnight, until the optical density reached 0.8 at 600 nm—it was prepared as bacteria for inoculation. Then 0.5 ml of bacteria for inoculation were added to a 250 ml conical flask containing 50 ml of specific medium, namely Basic Liquid Media (BLM) for cellulase production; this media consisted of (g/l): glucose 0.5, peptone 0.75, FeSO_4_ 0.01, KH_2_PO_4_ 0.5, MgSO_4_ 0.5 and 0.25 g from each agriculture wastes added individually. The substrate served as the favorable source for higher cellulase enzyme activity was considered as suitable substrate for further studies. The inoculation medium was prepared and shaken at 180 rpm for 48 h at 37 °C. The culture medium was then centrifuged for 20 min at 4 °C and 8000 rpm.The supernatants were recovered as a source of the crude enzyme to determine the cellulase activity, the method was implemented properly as described by Lingouangou et al. [54]**.** Cellulase activity was measured following standardized procedure of Smogyi by estimating reducing sugar content [55]. In brief, 0.5 ml of cell free supernatant was taken as crude enzyme to form a reaction mixture with 1 ml of 1% (w/v) carboxymethyl cellulose (CMC) in 50 mM sodium phosphate buffer, pH 6.0 for 10 min at 50 °C. The reaction was stopped by adding 1 ml Somogyi copper reagent and boiling for 20 min in a water bath. The reaction mixture was first cooled down and then received 1ml of Nelson reagent and completed to 25 ml with distilled water [56]. The density of the developed color was measured spectrophotometrically at 660 nm against blank—containing all the reagents except crude enzyme. One unit of enzyme activity is defined as the amount of enzyme that liberates 1µ mole of reducing sugar, with mannose as a standard per min per ml of culture filtrate. The blank coincided with the experiment in which the culture filtrate was boiled before the reaction.

### Molecular identification of the most efficient bacterium in CMCase production

#### Bacterial DNA purification and PCR amplification

The most cellulase-productive strain was designated as elh1. A single colony of elh1 was incubated overnight in nutrient broth (NB) at 30 °C. The bacterial culture was then centrifuged to obtain the pellet, which served as the source of genomic DNA. The GeneJET Genomic DNA Purification Kit (Thermo Scientific^™^, USA) was used to extract the genomic DNA. The 16S rRNA gene was amplified using universal bacterial primers: 8f forward and 1429R reverse primers. The sequences of these primers are as follows: 5'-AGAGTTTGATCMTGGCTCAG' and 5'-TACGGYTACCTTGTTACGACTT'. The PCR reaction mixture (50 µL) was prepared as follows: 2 µL each of the forward and reverse primers (at a concentration of 10 pmol), 25 µL of 2 × EmeraldAmp^®^ GT PCR Master Mix, 4 µL of bacterial DNA template, and PCR-grade water adjusted to 50 µL. The PCR conditions were as follows: a three-minute initial denaturation at 95°C, followed by 35 cycles of denaturation for 30 s at 95 °C, annealing for 30 s at 50 °C, and elongation for one minute at 72 °C. A BioRad T100 Thermal Cycler was used for the last extension phase, which was carried out for ten more minutes at 72 °C. The target fragment was visualized on an ethidium bromide-stained 1.2% agarose gel. Purification of the gel was performed using the GeneJET Gel Extraction Kit (Thermo Scientific^™^, USA). The sequencing of the target gene was carried out by Macrogen Inc. (Amsterdam, the Netherlands) using the Applied Biosystems 3730XL sequencer.

#### Phylogenetic analysis

BioEdit 7.1.10 was used to assemble the sequences [57]. The sequences were compared with those in the GenBank database (http://www.ncbi.nlm.nih.gov/blast) to identify comparable species. A collection of 16S rRNA genes from 13 related species available in the GenBank database was employed to construct a phylogenetic tree. Multiple sequence alignments were carried out using the MUSCLE algorithm [58] in MEGA11 [59]. The evolutionary history was inferred using the neighbor-joining method [60] with a 1000-bootstrap runs [61], and evolutionary distances were computed using the Jukes and Cantor method [62].

### Optimization of CMCase production conditions

#### One-variable-at-a-time approach (OVAT)

In this experiment, BLM medium components were changed to enhance the cellulase activity by identification of the key components of the medium. Each experiment was replicated three times to minimize variation. Results were expressed as mean ± standard deviation. Experimental factors included varying incubation durations (24, 48, 72, 96, and 120 h); rice peel, identified as the most effective waste for cellulase production, was incorporated at different concentrations (g/100 ml: 0.5, 1.0, 1.5, 2.0, 2.5, 3.0, 3.5, and 4.0); nitrogen sources (yeast, peptone, corn steep, casein, (NH_4_)_2_SO_4_, and urea) were added at a concentration equivalent to that in the original culture medium; sugar sources (sucrose, xylose, glucose, galactose, lactose, dextrose, and fructose) were included at a concentration of two grams per liter. Also, the resulting most efficient organic nitrogen source, urea, was then used at diverse concentrations (0.05,0.15,0.25, 0.35, 0.5,0.6,0.7 and 0.8 gm g/L) in the culture medium to determine the optimal concentration for cellulase production. Also, dextrose as the most efficient sugar for cellulase production was utilized in different concentrations (g/100 ml): 0.2, 0.3, 0.4, 0.5, 0.6, 0.7, 0.8. All experiments were supplied with inoculum size of 1% (v/v) and established at 37 °C, pH 7, for 48 h. Each parameter was examined individually while maintaining all other conditions at their optimal levels. At the conclusion of the specified incubation period for each parameter, approximately 5 ml of the incubated medium was collected and subjected to centrifugation at 4 °C for 10 min at 7000 rpm. The resulting supernatants were then utilized for the determination of cellulase activity via the aforementioned assay method. Each parameter was tested in triplicate. This method was implemented following that of Fouda et al. [28]. Results were expressed as mean ± S.D.; preliminary screening results will serve as a basis for the RSM experiment.

### Statistical optimization of rice peel based‑medium for CMCase production

#### The Plackett–burman design (PBD)

The relative significance of the different components within the medium was assessed and evaluated using the Plackett–Burman experimental design. Seven key factors with the greatest impact on enzyme production were selected for optimization. In order to pinpoint the critical factors that promote increased cellulase production, a total of seven variables were examined carefully, encompassing six components of the media (rice peel (X1), Urea (X2), Dextrose (X3), MgSO_4_.7H_2_O (X4), KH_2_PO_4_ (X5), and CaCl_2_ (X6)), and one cultivation parameter (inoculum size (X7)). Eleven experiments were conducted, with each variable designated and utilized at either high (+) or low (−) concentration. [30]. As indicated in Table 1, Each row denotes a test iteration, while each column signifies the concentration of a distinct independent variable. The experimental data were fitted in the following linear regression Eq. (1):

1 $$ Y = \beta 0 + \sum\nolimits_{i = 1}^{n} {\beta iXi} $$

where Y represents the response for cellulase enzyme activity (U/ml), β0 is the model intercept, βi is the linear coefficient, and Xi is the level of the independent variable.

The effect of each variable was determined by the following Eq. (2)

2 $$ {\text{E }}\left( {{\text{Xi}}} \right) \, = { 2}\left( {\sum {{\text{Mi}}} + \, - \, \sum {{\text{Mi}} - } } \right)/{\text{N}} $$

where Mi + and Mi– represent the response for cellulase enzyme activity (U/ml) from trials in which the Mi + and Mi– are the activity percentage in trials. The independent variable (Xi) was varied at both high and low concentrations, denoted as such, with N representing the total number of trials. The standard error (SE) was computed using the square root of each effect’s variance. The significance of each concentration effect was determined through Student’s t-test, with a significance level set at p < 0.05, as per Eq. (3).

3 $$ {\text{t }}\left( {{\text{Xi}}} \right) \, = {\text{ E}}\left( {{\text{Xi}}} \right)/{\text{SE}} $$

The effect of variable Xi is denoted by E(Xi). Regression analysis of the experimental data was conducted using SPSS Version 15.0.

### Box–Behnken design (BBD)

Based on the findings from the initial one-factor-at-a-time (OFAT) experiments, the experimental setup and statistical study were executed using Design-Expert software, trial version 11.0 (Stat-Ease Inc., Minneapolis, USA). The BBD (Box-Behnken design) three-level, three-factor design was used, comprising of 17 experimental runs. In this study, three variables were included: (X_1_) rice peel, (X_2_) Urea, and (X_3_) Dextrose. Each variable varied over three coded levels of−1, 0, + 1; the designation of “high” was represented as (+1), “medium” as (0), and “low” as (−1) [63]. The generalized second-order polynomial model employed in the RSM is presented in Eq. (4):

4 $$ {\text{Y}}_{{{\text{Activity}}}} = \beta 0 \, + \beta {\text{1X1 }} + \beta {\text{2X2 }} + \beta {\text{3X3 }} + \beta {\text{11X12 }} + \beta {\text{22X22 }} + \beta {\text{33X32 }} + \beta {\text{12X1X2 }} + \beta {\text{13X1X3 }} + \beta {\text{23X2X3}} $$

where Y represents the predicted response [i.e. CMCase activity (U/ml)]; β0 is the model constant; X1, X2, and X3 are the coded input variables which infuence the response variable; β1, β2, and β3 are the linear coefficients; β12, β13, and β23 are the cross-product coefficients; β11, β22, and β33 are the quadratic coefficients. All experiments were performed three times independently. All experimental outcomes and the corresponding standard deviations were derived from the mean of triplicate trials. A paired t-test was performed using the Statistical Package for Social Sciences (SPSS) for Microsoft Windows Version 15.0 to determine the actual and anticipated responses.

### Statistical evaluation of the model

The model underwent statistical analysis to evaluate the analysis of variance (ANOVA). The significance of the model equation was determined by Fisher’s test value, while the proportion of variance explained by the model was indicated by the estimation of multiple coefficients for each variable. Quadratic models were visualized using contour plots (3D), and response surface curves were generated using Design-Expert software, trial version 11.0 (Stat-Ease Inc., Minneapolis, USA). The predictive accuracy of the polynomial model equation was assessed using the coefficient of determination R2 and adjusted R2.

### Primer Design and egl Gene amplification

The full-length egl gene sequence, annotated as an endo-1,4-β-glucanase, was retrieved from the genomic DNA of *Bacillus amyloliquefaciens,* Reference genome ASM1939692v1. We have performed an automated Uniprot Blast using this sequence as query, which recorded 98.8% identity with uniprotkb/P07983/entry (Endoglucanase from *Bacillus subtilis* as a reviewed entry). Additionally, the egl gene sequence retrieved from EMBL (https://www.ebi.ac.uk/ena/browser/api/embl/MK675502) under the MK675502.1 entry was utilized for the primer design. Primer3 program (https://www.bioinformatics.nl/cgi-bin/primer3plus/primer3plus.cgi) was utilized for this purpose. The sense primer, egl-F, has a nucleotide sequence of 5'-GAGGCTCATGAAACGGTCAATCTCTATT-3', emphasizing a SacI recognition site, and the antisense primer, egl-R, has a sequence of 5'-GCATGCCTAATTTGGTTCTGTTCCCCAA-3', emphasizing an SphI recognition site. PCR-based amplification of the egl gene was carried out using Phusion High-Fidelity DNA Polymerase from Thermo Scientific^™^ according to its provided protocol, with an annealing temperature of 50 °C. Phusion DNA Polymerases exhibit robust performance, short protocol times, tolerance to PCR inhibitors, and produce higher yields with lower enzyme amounts compared to other DNA polymerases.

### Gene cloning and transformation

A 1.2% agarose gel was used to electrophorese the PCR product, and the band believed to include the desired gene was removed. The GeneJET Gel Extraction Kit from Thermo Scientific (Waltham, Massachusetts, United States) was used for processing. A 1500 bp fragment compatible with the ORF of the egl gene was isolated from the agarose gel using the extraction kit. The purified product was then ligated into the pGEM-T Easy-cloning vector using T4 DNA Ligase (Thermo Scientific™) following the recommended procedures. The ligated product was transformed into competent *E. coli* DH5α cells (100 µL) prepared according to the method outlined by Chung et al. [64]. The recombinant plasmid was introduced into *E. coli* DH5α cells via the heat shock method: the mixture was immediately added to 500 µL of LB media in a sterilized 2 ml Eppendorf, which was shaken for two hours at 37 °C to permit the expression of antibiotic ampicillin gene. Subsequently, an appropriate volume was plated on LB agar plates containing 0.5 mM IPTG, 50 µg/ml ampicillin, and 40 µg X-gal, followed by overnight incubation at 37 °C. White colonies were selected and examined for the presence of the recombinant vector. Purification of recombinant vector was accomplished using Plasmid Miniprep Kit. Standard protocols for restriction endonuclease digestions, agarose gel electrophoresis, DNA purification from agarose gels, DNA ligation, and other cloning-related techniques were employed as described by Sambrook and Russell [65]. Two techniques were used to evaluate the integrity of the recombinant plasmids: colony PCR and double digestion of the recombinant vector using SacI and SphI restriction enzymes. The ORF of egl gene was subjected to DNA sequencing and the recombinant vector was designated as pGEM-egl. The positive transformant cells were subsequently transferred to LB agar media supplemented with 1% CMC (w/v) to observe clear zone. Then approximately 0.5 ml of overnight culture derived from positive transformant cells, exhibiting an optical density of 0.8 at 600 nm, was introduced into a 250 ml conical flask containing 50 ml of Basic Liquid Media (BLM) supplemented with 0.25 g of rice peel. The flasks were incubated for 72 h at 37 °C with continuous shaking at 180 rpm. After incubation, the culture medium was centrifuged for 20 min at 8000 rpm and 4 °C.The resulting supernatant was collected as the crude enzyme source, which was utilized for cellulase activity determination. The same procedure was followed for the not-transformed parent *E. coli* strain.

### DNA sequencing and in silico analysis of egl gene

The nucleotide sequence was determined using the dideoxynucleotide chain termination method with the specific primers, egl-F and egl-R [66]. Following sequencing, editing was conducted to correct inaccuracies and trim unreadable portions at the 3' and 5' ends using BioEdit version 7.0.2 software. The edited sequence was compared against the NCBI nucleotide database (https://blast.ncbi.nlm.nih.gov) to determine its taxonomic identity, and a unique accession number was assigned to the sequence. To identify homologous proteins, the deduced amino acid sequence of the egl gene was aligned with the UniProt protein database (https://legacy.uniprot.org/align/). The ESPript 3.0 program was used to incorporate superimposed predicted secondary structures into the alignment, providing insights into the structural features of the egl gene product [67]. The Conserved Domain Database, accessible via the NCBI website (https://www.ncbi.nlm.nih.gov/Structure/cdd/wrpsb.cgi), was searched to investigate the conserved domains. This analysis helped to determine the functional domains and potential catalytic regions of the endoglucanase enzyme. Additionally, version 6.0 of the SignalP software (http://www.cbs.dtu.dk/services/SignalP/) was employed to analyze the N-terminal signal peptide, which is crucial for the secretion of the enzyme [68]. To investigate the evolutionary relationships between the deduced egl amino acid sequence and other homologous proteins, the MEGA11 software was utilized [59]. The MUSCLE algorithm was used to conduct multiple sequence alignment [58], and the neighbor-joining method was applied to infer the phylogenetic relationships [60]. A 1000-replicate bootstrap test was conducted to assess the statistical support for the branching patterns in the phylogenetic tree [61].

### Template search, comparative modeling and model confirmation

The Expasy translate tools, accessible at web.expasy.org/translate/, were used to translate nucleotide sequences and align deduced amino acid sequences. A homology model was constructed using SWISS-MODEL [69–72]. The translation of the cellulase encoded by the egl gene provided the protein sequence input for the model.In order to guarantee adequate coverage of the query sequence and sequence identity within the template library, a template search was carried out on the SWISS-MODEL web server using Blast and HHBlits in order to construct the model. The target sequence was compared to the primary amino acid sequence in the SMTL database using the BLAST algorithm [73]. To choose the most trustworthy 3D structure, it is essential to evaluate the values of both the Qualitative Model Energy Analysis (QMEAN) and Global Model Quality Estimation (GMQE) [75]. more numbers, which usually lie between 0 and 1 for GMQE values, suggest greater reliability of the projected structure. Higher reliability is indicated by a QMEAN value below 4.0 in the model’s quality assessment [76].

### Structure validation of modelled protein

To validate the structure of the modeled protein, we ran the following tools: the ProSA server (https://prosa.services.came.sbg.ac.at/prosa.php) and SAVES version 6.0 (Structure Analysis and Verification Server). SAVES v6.0 includes five programs: PROCHECK [53], VERIFY-3D [77], and ERRAT [78]. All these tools were utilized to assess the 3D protein models, evaluate the quality of the model by examining the allowed and disallowed regions on the plot, and determine the similarity of the model to native nuclear magnetic resonance/X-ray crystal structures [79]. This comprehensive validation process ensures the accuracy and reliability of the modeled protein structure by assessing its compliance with established structural standards and principles. In particular, the Ramachandran plot and statistics were used to examine the permitted and prohibited areas on the plot in order to assess the quality of the model.A Z score value was produced by the ProSA web service [79], which provided information about the model’s general quality and similarity to native nuclear magnetic resonance/X-ray crystal structures. This comprehensive validation process ensures the accuracy and reliability of the modeled protein structure by assessing its conformity to established structural standards and principles.

### Alignment of the CMCase model and the template structure

The alignment between the modelled CMCase structure and the template structure was implanted by the PyMOL molecular viewer [80] and proximity of the carbon atoms also illustrated. The root mean square deviation (RMSD) is the main metric used to measure the difference in carbon atom locations between the template and model. The degree of structural similarity between two entities is larger when the RMSD is lower, almost nil [81].

### Molecular docking

A computational analysis was conducted using the Molecular Operating Environment (MOE) software Version 2015.10 [82]. Preparation of the modeled protein, CMCase, and ligand Carboxymethyl cellulose (PubChem CID 24748) involved removing water molecules and adding hydrogen atoms. Subsequently, a molecular database (MDB) file containing the protein's 3D structure attached to the ligand for docking simulations was created [83]. Docking poses were chosen based on the ratings and root mean square deviation values. Amino acids in the active site were determined. Chimaera was used to visualize the receptor-binding site and analyze ligand-receptor interactions [84], with a focus on key amino acid residues involved in hydrogen bonding. To evaluate the interactions, established criteria for molecular interactions were followed [85].

### Data analysis

Data processing was done using Design-Expert software, trial version 11.0 (Stat-Ease Inc., Minneapolis, USA).

## Data Availability

All data generated or analyzed during this study are included in this article.
